# Comprehensive Metabolomic Fingerprinting Combined with Chemometrics Identifies Species- and Variety-Specific Variation of Medicinal Herbs: An *Ocimum* Study

**DOI:** 10.3390/metabo13010122

**Published:** 2023-01-13

**Authors:** Abhishek Kumar Rai, Samreen Khan, Akhilesh Kumar, Basant Kumar Dubey, R. K. Lal, Ashutosh Tiwari, Prabodh Kumar Trivedi, Christopher T. Elliott, Ratnasekhar Ch

**Affiliations:** 1 Metabolomics Lab, CSIR—Central Institute of Medicinal & Aromatic Plants (CIMAP), Lucknow 226015, India; 2 Department of Genetics and Plant Breeding, CSIR—Central Institute of Medicinal & Aromatic Plants (CIMAP), Lucknow 226015, India; 3 Department of Biotechnology, CSIR—Central Institute of Medicinal & Aromatic Plants (CIMAP), Lucknow 226015, India; 4 Academy of Council of Scientific and Industrial Research (ACSIR), Gaziabad 201002, India; 5 School of Biological Sciences, Queen’s University Belfast, Belfast BT9 5DL, UK; 6 School of Food Science and Technology, Faculty of Science and Technology, Thammasat University, 99 Mhu 18, Phahonyothin Road, Khong Luang 12120, Pathum Thani, Thailand

**Keywords:** metabolomic fingerprinting, *Ocimum* species and varieties, medicinal herbs, chemometrics

## Abstract

Identification of plant species is a crucial process in natural products. *Ocimum*, often referred to as the queen of herbs, is one of the most versatile and globally used medicinal herbs for various health benefits due to it having a wide variety of pharmacological activities. Despite there being significant global demand for this medicinal herb, rapid and comprehensive metabolomic fingerprinting approaches for species- and variety-specific classification are limited. In this study, metabolomic fingerprinting of five *Ocimum* species (*Ocimum basilicum* L., *Ocimum sanctum* L., *Ocimum africanum Lour.*, *Ocimum kilimandscharicum Gurke.*, and Hybrid Tulsi) and their varieties was performed using LC-MS, GC-MS, and the rapid fingerprinting approach FT-NIR combined with chemometrics. The aim was to distinguish the species- and variety-specific variation with a view toward developing a quality assessment of *Ocimum* species. Discrimination of species and varieties was achieved using principal component analysis (PCA), partial least squares discriminate analysis (PLS-DA), data-driven soft independent modelling of class analogy (DD-SIMCA), random forest, and K-nearest neighbours with specificity of 98% and sensitivity of 99%. Phenolics and flavonoids were found to be major contributing markers for species-specific variation. The present study established comprehensive metabolomic fingerprinting consisting of rapid screening and confirmatory approaches as a highly efficient means to identify the species and variety of *Ocimum,* being able to be applied for the quality assessment of other natural medicinal herbs.

## 1. Introduction

Plants are effective biochemists, producing potent biomolecules used since ancient [1] times for curing a range of diseases in the form of traditional systems medicine [2]. More than half of the global population is dependent on traditional medicines for health care, as cited in a World Health Organisation report [3]. The global demand for medicinal plants has increased considerably due to their proven effectiveness in treating various diseases [4]. The beneficial medicinal effects of plant materials result from the combination of secondary metabolites present in the plants, according to the specific chemical structure of each biochemical active compound [5]. The medicinal activity obtained from these wide varieties of different phytochemicals is plant, tissue, species, or variety specific or taxonomy specific. In many parts of the world, medicinal herbs play a substantial part in diets as they are key ingredients in many foods, beverages, pharmaceuticals, and cosmetics [4,6]. 

*Ocimum*, a genus of aromatic annual and perennial herbs in the family Lamiaceae, has been used for thousands of years due to its diverse medicinal properties [7]. It is widely distributed over temperature zones (warmer regions) of the world [8]. It has been used for antioxidant and anti-inflammatory purposes as its extracts protect nerves and tissues by preventing the generation of free radicals [7,9]. Furthermore, this plant is well known for its anti-depressant and anti-aging properties due to the presence of a wide variety of secondary metabolites [10]. Moreover, this plant is most popular in the culinary world, and it is widely used in cooking, in many types of cuisines, and it is also used in food flavouring and preservation [11,12]. *Ocimum* is known as the queen of herbs and is prominently featured in various cuisines across the world including Italian, Vietnamese, Chinese, Thai, Laotian, and Indian [11,12,13]. This plant contains a wide variety of antioxidants and has anti-microbial properties, due to which it is widely used in food and beverages [7,14]. Further, this is an aromatic and medicinal plant with high economic value that is used in the pharmaceutical and aroma industries [15]. Compared to most other herbs, the taxonomy of the *Ocimum* genome is considered to be very complex. More than 100 species have been recognised within the genus [16]. The quality control of such medicinal plants is extremely challenging as it is not only limited to the botanical level but also given that there are significant variations of chemical profiles within the same species [17]. Indeed, the secondary metabolite expression in a given plant is a function of biotic, abiotic, and genetic factors that specify species, varieties, and cultivars [18]. Effective identification systems and robust methods for the species and variety classification of medicinal plants are needed. 

Most of the taxa classification is based on morphology (macro and microscopic identification) and the colour of leaves [19]. These morphological properties frequently depend on a range of environmental conditions, leading to ambiguity in the classification within the genus, and there is enormous variation in the shape and colour of the leaves from different species and within the varieties. In addition to these techniques, DNA-based methods have been proven to be robust for the unambiguous identification of the medicinal plant genus; however, these methods fail to identify the mixing of species that is responsible for the lowering of the quality of medicinal plant products [16]. Furthermore, classification based on volatile oil composition requires the distillation and fractionation of oils, and the chemotype classification based on only one major volatile oil is erroneous as one plant may contain two or more chemical compounds in nearly equal amounts [15,20,21]. In addition, the overall oil profile of major constituents above the fixed threshold 20% of total essential oil content should be considered. 

Plant-metabolome-based fingerprinting methods are gaining more attention to address the pitfalls of the previously mentioned techniques to determine metabolite fingerprints for species-specific and variety-specific variation [22,23,24,25]. Nuclear magnetic resonance (NMR) spectroscopy and mass spectrometry (MS) are commonly used metabolomics platforms. Although NMR is a non-destructive and straightforward procedure, limited number of metabolites are identified due to the complex spectra of wider plant metabolome consisting of primary and secondary metabolites [26]. The separation and identification of complex spectra of metabolite profile obtained by MS-based analytical platforms are improved by the coupling to a range of separation techniques such as gas chromatography and liquid chromatography, the latter of which not only enhances the separation efficiency but also improves the performance of MS. Liquid chromatography/gas chromatography coupled to mass spectrometry have become central platforms in metabolomics studies, and the recent application of these metabolomics platforms, specifically in natural products and plant sciences, has gained more attention [27]. The resolution of hundreds of metabolites with analyte-specific detection and the ability to identify unknowns make these MS-based platforms suitable tool for fingerprinting studies. In addition to these, spectroscopic methods are gaining more attention for metabolic fingerprinting as they are quick and non-destructive [28]. Spectroscopy has been used as a herbs and spices screening tool. For example, Fourier transform infrared (FT-IR) has been used in a range of medicinal herbs and natural medicine. Recently, medicinal herb metabolome fingerprinting has been used for the assessment of various species and authenticity [28]. A significant number of studies have been devoted to describing *Ocimum* essential oils [21,29,30], but relatively few studies have been devoted to *Ocimum* leaves as there is a lack of a comprehensive and rapid fingerprinting approach [16,31,32]. Given the fact that *Ocimum* leaves are used for various medicinal uses and to add distinctive flavours and aromas for the food and pharmaceutical industries, there is a need for comprehensive and rapid metabolomic fingerprinting of *Ocimum* species and varieties.

In the present study, an untargeted comprehensive multi-metabolomics approach was used to investigate the phenotype of five different *Ocimum* species: *Ocimum basilicum* L*. (O. basilicum)*, *Ocimum sanctum* L. *(O. sanctum)*, *Ocimum africanum Lour. (O. africanum)*, *Ocimum kilimandscharicum Gurke. (O. kilimandscharicum)*, and Hybrid Tulsi, which are the major species used globally. GC-MS- and LC-MS/MS-based metabolomics combined with multivariate pattern recognition analysis was used to identify fingerprints with potential marker metabolites of *Ocimum* species. The abundance of nine chemical classes of metabolites were compared between these samples. Further, FT-NIR-based metabolic fingerprinting combined with one-class classifier models, DD-SIMCA and K-nearest neighbor, were used to classify the *Ocimum* species and varieties. Moreover, eight different varieties from *Ocimum* species were studied. The aim of the study was to identify metabolite markers through comprehensive and rapid metabolomic fingerprinting of *Ocimum* species and variants because they are utilised for numerous therapeutic purposes and to offer distinctive flavour, fragrance, and aroma to the food and the pharmaceutical industries.

## 2. Materials and Methods

### 2.1. Plant Materials

The leaves of five different *Ocimum* species, namely, *O. basilicum*, *O. sanctum*, *O. africanum*, *O. kilimandscharicum*, and Hybrid Tulsi with different varieties, were collected from the research field of the CSIR—Central Institute of Medicinal and Aromatic Plants (CIMAP) in October 2021 (Table S1). CSIR-CIMAP has a history of *Ocimum* cultivation for more than fifty years, and all the *Ocimum* varieties planted have been confirmed by expert botanists. *Ocimum* plants with similar growth without diseases were randomly selected. It is important to note that fresh *Ocimum* leaves are rarely available on the markets and are usually dried and then packaged for storage, transportation, and processing. It is also important to note that freeze-drying (lyophilisation) is an effective drying process without compromising the quality [33]. Therefore, after harvest, the fresh *Ocimum* leaves were rinsed with water and subjected to freeze-drying. The dried *Ocimum* leaves were ground to a powder, passed through a mesh, sealed in tubes, and then stored at −80 °C until further use.

### 2.2. Sample Preparation and LC-MS/MS Conditions

The dried powder of *Ocimum* leaves of each sample was weighed precisely and extracted with 1.5 mL of 70% methanol (HPLC grade, Sigma Aldrich, St. Louis, MO, USA) containing U^13^C_6_ glucose (an internal standard, Cambridge Isotope laboratories, Andover, MA, USA). Briefly, the extraction process involved the sonication of samples (using an ultrasonic water bath) for 10 min under ice-cold conditions, a further vortex mix for 30 min, followed by centrifugation for 20 min at 13,523 rcf. The supernatant was collected into fresh tubes. The above extraction process was repeated twice, and the combined extracts were concentrated using lyophilisation (Alpha 2-4 LD plus, Christ, Germany). The dried samples were reconstituted with 400 µL of 70% methanol (HPLC grade) for the LC-MS analysis. 

The UPLC analysis was performed on an instrument of the Agilent 1290 series (Agilent Technologies, Santa Carla, CA, USA), composed of a Binary pump (G7120A), autosampler (G7129A), Column oven (G7130A), and diode array detector (G4212B). The UPLC separation was accomplished on an Acquity UPLC BEH C18 1.7 µm 2.1 mm× 150 mm (Waters, Milford, MA, USA) operated at 40 °C. Gradient elution was achieved using two solvents: 0.1% (*v*/*v*) formic acid aqueous solution (A) and 0.1% (*v*/*v*) formic acid in acetonitrile (B) at a flow rate of 0.2 mL/min. The 30 min UPLC gradient elution program was as follows: (i) 75%, from 0 to 20 min (B); (ii) 75%, from 25 min (B); (iii) 5%, from 26 to 30 min (B) of total run time; the injection volume was 2 µL. The MS analyses were performed on a QTOF–MS/MS instrument of the Agilent 6545 series (G6545A), connected with an Agilent 1290 UPLC (Agilent technologies, Santa Clara, CA, USA) through a dual AJS ESI interface. Nitrogen was used as the drying and collision gas in the ESI source. The ion source parameters were as follows: drying gas flow rate, 10 mL/min; heated capillary temperature, 330 °C; nebuliser pressure, 35 psi; VCap, fragmentor, skimmer, and octopole RF peak voltages set at 4000, 180, 45, and 750 V, respectively. The detection was carried out in positive and negative electrospray ionisation modes, and spectra were recorded by MS scanning in the range of *m*/*z* 80–1500. The MS/MS analyses were carried out by data-dependent acquisition, and the collision energy was set at 10–30 eV. Mass Hunter software version B.07.00 (Agilent Technology) was used to control the LC-MS/MS system, data acquisition, and processing. 

### 2.3. Sample Preparation and GC-MS Conditions

The lyophilised samples of different varieties of *Ocimum* were stored in air-tight containers at −80 °C after being treated with liquid nitrogen to prevent metabolic activity. Before extraction, dried leaves were ground using a mortar and pestle. The dried powder of the leaves of each sample was weighed precisely and we added 1.5 mL of 70% methanol (HPLC grade) containing 10 µg/mL uniform 13 C6 glucose (an internal standard for relative quantification); following this, the mixture was vortexed vigorously for 10 min and then sonicated for 30 min at 20 using an ultrasonic water bath (53 kHz). Extracts were then vortexed vigorously for 40 min and centrifuged at 13,523 rcf for 20 min at 21 °C to remove plant debris. The supernatant was collected and transferred into a clean tube, and after the extraction of each sample, the supernatant was concentrated by lyophilisation. QC samples were prepared by pooling aliquots (10 µL) from all extracted samples. QC samples were dried again completely using the lyophiliser. The dried samples were resuspended in 60 μL of methoxyamine hydrochloride solution in pyridine (20 mg/mL), then vortexed for thorough mixing and thereafter incubated in a thermomixer for 2 h at 37 °C with 60 rcf. MSTFA (110 μL) (Sigma Aldrich, St.Louis, MO, USA) was then added to each sample, and then we vortexed all the samples and incubated in a thermomixer for 30 min at 37 °C with 60 rcf and injected the samples into GC-MS for analysis.

The GC-MS analysis was performed using an Agilent Technologies 7980A gas chromatography system with the 5977A mass selective detector (Santa Carla, CA, USA). The HP5-MS column with a dimension of 30 mL × 250 µm with film thicknesses of 0.25 µm was used for obtaining the peak separation in the chromatogram. Helium in a split ratio of 3:1 and a flow rate of 1.5 mL/min was used as the carrier gas. The running condition for the samples was 70 °C for 2 min as initial hold and heating ramp of 12.5 °C/min until the temperature reached 295 °C, and finally, with a ramp rate of 25 °C/min, the temperature reached 320 °C, with 5 min as a final hold. Mass spectrometry was conducted at 230 °C as a transfer line and ion source temperature, while 150 °C was the quadrupole temperature, 70 eV the ionisation potential, and 40 to 700 the atomic mass units scan range. Identification of the compounds was performed by comparing their retention indices with those of known compounds obtained by injecting a mixture of standards containing a homologous series of C7–C30 alkanes analysed in the same column under the same chromatographic conditions.

### 2.4. FT-NIR Spectroscopy Analysis

FT-NIR spectra were acquired using the ANTARIS II FT-NIR spectrometer (Thermo Scientific Co., Waltham, MA, USA) equipped with an interferometer and an integrated sphere. Approximately 1 g of weighed powdered samples were placed in glass vials, and spectra were recorded in the range of 10,000–4000 cm^−1^ by using 64 scans. The FT-NIR raw datasets were measured with a spectral resolution of 4 cm^−1^, resulting in 1557 variables. The FT-NIR reflectance spectra were expressed as log (1/R), where R is the reflectance. In order to remove any systematic variation in the model, sample spectra were randomly generated. All spectral measurements were carried out at room temperature (26 ± 1 °C).

### 2.5. Data Processing and Analysis

The raw LC-MS and GC-MS files were processed using Agilent Mass Hunter software and Progenesis. Automated peak detection, retention time alignment, and peak matching were performed, and the data matrix consisted of Rt/(*m*/*z*); samples and intensities were further used for statistical analysis [24,34]. Total area normalisation of samples and scaling were performed to make features more comparable in magnitude to each other. Multivariate statistical analysis was performed by using the online platform R. Principal component analysis (PCA) was performed in order to have a better visualisation of all the information contained in the dataset. To further identify the differential metabolites that account for the separation between groups, supervised PLS-DA was used [35]. The developed PLS-DA model was validated using the leave one out cross validation method, and its quality was assessed on R^2^ and Q^2^ scores [36]. Furthermore, this model was validated using 1000 times permutation tests [36]. The PLS-DA model generates variable importance in projection (VIP) scores. Metabolites with VIP values greater than 1 were identified as potential differential compounds [37]. The unpaired *t*-test was used to perform univariate analysis. Hierarchical cluster analysis was performed for identifying relatively homogenous clusters of various sample groups on the basis of measured characteristics. For LC-MS, metabolites with mass error less than 5 ppm were considered, and further MS/MS identification of metabolites were performed. These were identified using Metlin, HMDB, PubChem, and KEGG libraries and using an inhouse library. An Automated Mass Spectral Deconvolution and Identification System (version 2.0, NIST, Gaithersburg, MD, USA) was used to perform deconvolution, which enabled us to extract the clean mass spectra from a complex process and helps in the correct identification of the metabolites. The spectra obtained after deconvolution were compared with the pure spectra of standards, when available, and the spectra available, with the NIST library (a match quality of 90% minimum was used as a criterion (v 2.2 g distributed with NIST 2014, USA)) [38].

### 2.6. One Class Classification Model

MATLAB R2021a version 9.10 (MathWorks, Inc., Natick, MA, USA) was used for performing DD-SIMCA models [35]. This one class classifier method is meant to distinguish objects of one target class from all other objects and classes. R version 3.4.1 was used for performing K-nearest neighbour classification [39,40].

## 3. Results and Discussion

Plant species identification is one of the essential aspects. Identification of species or a variety of natural medicinal herbs is of great interest to select the correct plants with specific pharmacologically active secondary metabolite profiles, ensuring no adulteration along its complex production chain, and protecting the product’s commercial value. There is a growing body of evidence that metabolic fingerprinting can be used to identify the species/variety characterisation of natural medicines. Despite *Ocimum* being widely used across the world as a traditional medicine and in pharmaceutical and food industries to add a distinctive flavour and aroma to foods, the comprehensive and rapid metabolic fingerprinting of species and varieties of this herb has not been well defined. Therefore, the LC-MS, GC-MS, and FT-NIR-based comprehensive metabolic fingerprinting approach was employed to identify marker compounds and spectral fingerprints to classify samples on the basis of species/varieties.

### 3.1. LC-MS-Based Metabolic Profiling of Ocimum Leaves Identified Species-Specific Variation

**Figure 1:**
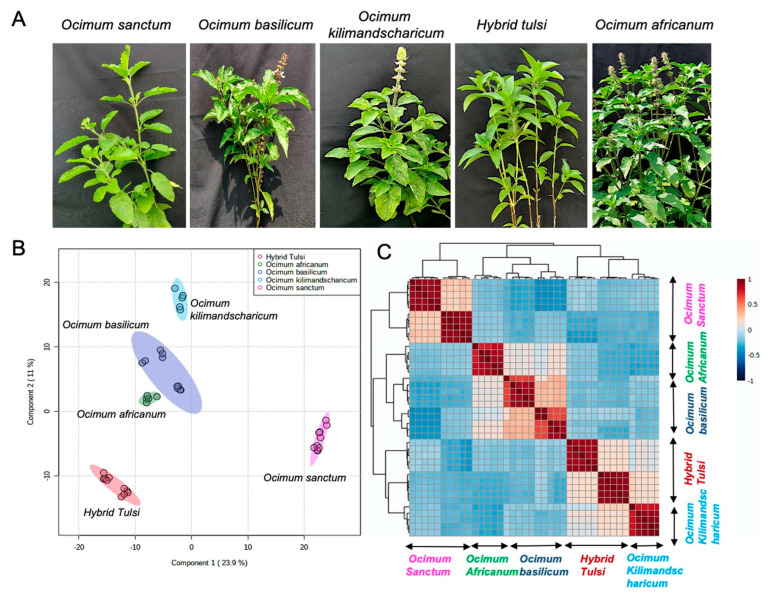
Differentiation of *Ocimum* samples from five different species according to their metabolite profiles. (**A**) Images of five different *Ocimum* species, namely, *O. sanctum*, *O. basilicum*, *O. kilimandscharicum*, Hybrid Tulsi, and *O. africanum.* (**B**) PLS-DA score plot for the first component (23.9%) vs. second component (11%), indicating discrimination between five different species of *Ocimum* samples acquired in LC-MS negative ionisation mode. (**C**) Pearson correlation of *Ocimum* samples from five different species.

Untargeted metabolite profiling using LC-QTOF-MS was performed in both ESI (+) and ESI (−) ionisation modes to cover the maximum metabolome of *Ocimum* samples. The corresponding QC plots for pooled quality control samples explained the tight clustering of samples, as shown in Figure S1A,B. The corresponding TIC of the acquired metabolite profiles in both ESI (−) and ESI (+) ionisation modes are shown in Figures S2 and S3, respectively. PCA was performed to gain a better visualisation of all the information presented in the datasets collected as it was possible to identify the differences among the various sample groups by projecting dataset objects into the space of the first few principal components. The unsupervised PCA obtained from the LC-MS spectra of all samples revealed the general structure of the complete dataset, in which the first two principal components accounted for 47.4% and 50.8% of the total variation in negative ionisation mode and positive ionisation mode, respectively (Figure S4A,B). Supervised PLS-DA was further performed to identify a small number of linear combinations of the original variables that described most of the variability of the metabolite profile of the five different species of *Ocimum* samples. As presented in Figure 1B and Figure S5, five different clusters corresponding to five different *Ocimum* species were identified in the PLS-DA scores plot for both (+) and (−) ionisation modes, in which the first two components cumulatively accounted for 34.9% variation in negative ionisation mode and 46.7% variation in positive ionisation mode, and with the first component explaining (23.9% for ESI negative mode; 36.1 for ESI positive mode) the variation between *Ocimum sanctum* samples from the remaining four species *O. basilicum*, *O. africanum*, *O. kilimandscharicum*, and Hybrid Tulsi and the second component explaining (11% for ESI negative mode; 10.6% for positive mode) the variation between Hybrid Tulsi and *O. sanctum* samples from *O. basilicum*, *O. africanum*, and *O. kilimandscharicum* samples.

**Figure 2:**
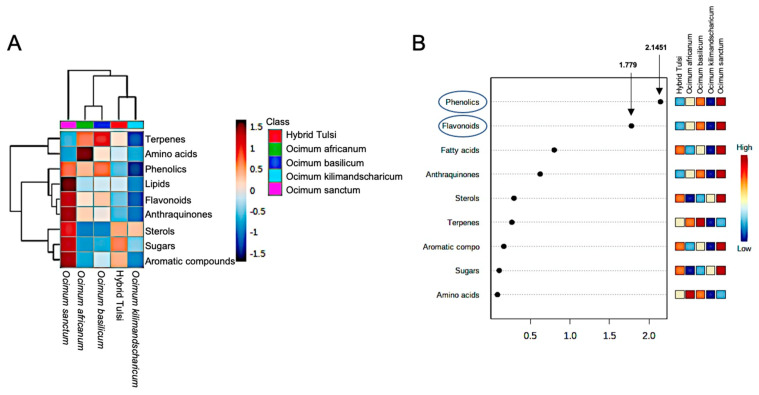
Classification of different metabolite classes in five different *Ocimum* species samples. (**A**) Quantitative analysis of nine metabolite classes in *Ocimum* species samples analysed using HCA. (**B**) Variable importance in projection of metabolite classes for the analysed five *Ocimum* species samples.

The corresponding loading plot responsible for the clustering of five different *Ocimum* species samples is shown in Figure S6. Tenfold cross-validation was further performed to find the predictive accuracy and fit of the polynomial model (Figure S6A,B). The PLS-DA cumulative values with Accuracy = 1.0, R2 = 0.9982, Q2 = 0.9949 for negative ionisation mode and Accuracy = 1.0, R2 = 0.9993, Q2 = 0.9965 for positive ionisation mode showed a good fit of the model. Furthermore, to assess the statistical significance of these deceptively highly predictive multivariate models, permutation tests were conducted by validating the models with 1000 permutation tests (Figure S7C,D). From the analysis of these distributions, the significance of the power of the optimal models to predict the *Ocimum* sample metabolite profile was determined to be *p* < 0.001. To correlate different *Ocimum* sample groups, Pearson correlations were performed between sample groups, and each *Ocimum* species sample group had a strong positive correlation with the corresponding sample groups (Figure 1C). In total, 3626 metabolite features were detected both in positive and negative ionisation modes. The identified metabolites were grouped into nine different chemical classes, including terpenes, amino acids, phenolics, lipids, flavonoids, anthraquinones, sterols, sugars, and aromatic compounds. Unsupervised hierarchical cluster analysis was performed for the quantitative analysis of nine metabolite classes from these five different *Ocimum* species samples (Figure 2A). *O. basilicum*, *O. africanum* and Hybrid tulsi, and *O. kilimandscharicum* were clustered together while leaving *O. sanctum* samples separately. There was a relatively higher quantity of terpenes and phenolics present in *O. basilicum* samples, while *O. africanum* contained a high concentration of amino acids. Conversely, high quantities of flavonoids, phenolics, anthraquenones, sterols, sugars, and aromatic compounds were present in *O. sanctum* samples. Importantly phenolic and flavonoid metabolite classes were responsible for the species-specific discrimination of *Ocimum* samples according to VIP values (Figure 2B). 

**Figure 3:**
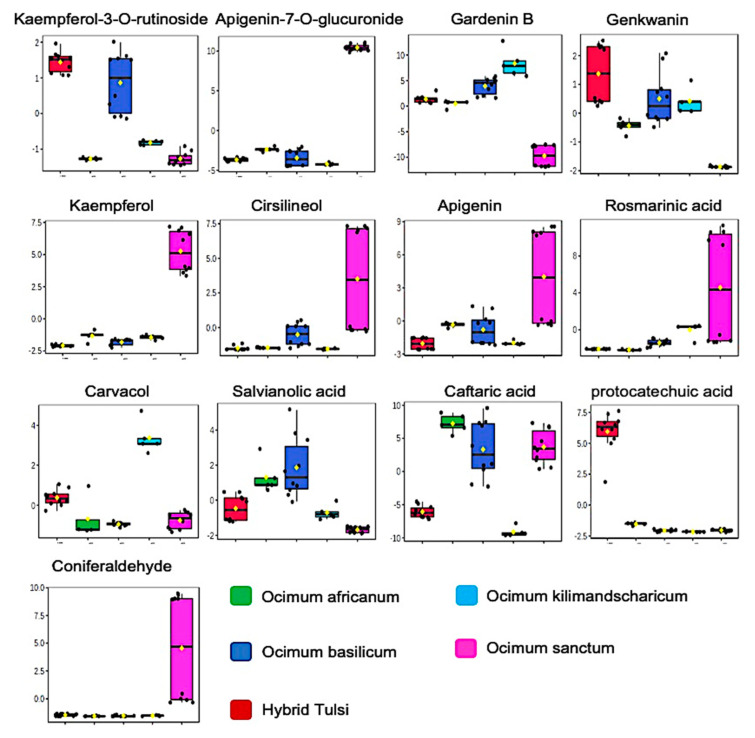
Box plot for discriminatory metabolite markers, identifying the differences in five different *Ocimum* species, namely, *O. sanctum*, *O. basilicum*, *O. kilimandscharicum*, Hybrid Tulsi, and *O. africanum.* Normalised intensities are presented in the *y*-axis.

In the present study, key flavonoids were identified as significant marker metabolites for the differentiation of species-specific *Ocimum* samples (Figure 3). These flavonoids are derived from the phenylpropanoid and flavone synthesis pathway [41]. The crucial function of flavonoids in plants are to protect them against various abiotic (salt, drought, UV radiation, and heat) and biotic stresses such as pathogen and herbivore attacks [42]. Plants use them as one of their defence mechanisms against oxidative damage and to combat fungal infections of plant leaves. Importantly, flavonoids quench the ROS by reducing the singlet oxygen levels, hindering of enzymes, lipoxygenase, xanthine oxidase, monooxygenase, and cyclooxygenase involved in ROS generation and help in the recycling of other antioxidants in plants. In the present study, high concentrations of apigenin apigenin-7-O-glucuronide, kaempferol, and cirsilineol were present in *O. sanctum* samples in comparison to other *Ocimum* species. Conversely, high concentration of gardenin B was present in *O. basilicum* and *O. kilimandscharicum* samples in comparison to other species. A relatively high concentration of kaempferol-3-O-rutinoside was observed in Hybrid Tulsi and *O. basilicum* and high concentration of kaempferol was observed in *O. sanctum*. 

Rosmarinic acid is an ester of 3,4-dihydroxyphenyllactic acid and caffeic acid, and both were found abundance in plants of the Lamiaceae family biosynthesised from phenylpropanoid- and tyrosine-derived pathways [43]. Plants use this antioxidant phenolic compound for their defence system [43]. In the present study, high concentrations of rosmarinic acid were present in *O. sanctum* samples in comparison to other species. Carvacrol is a monoterpene phenol with an abundant presence in many aromatic plants. This metabolite is produced through the methylerythritol (MEP) pathway obtained from isopentyl diphosphate (IDP) and dimethylallyl diphosphate (DMADP) in plastid [44]. In the present study, a relatively high concentration of carvacrol was present in *O. kilimandscharicum* samples in comparison to other *Ocimum* species. Salvianolic acid, a phenolic metabolite derived from the phenylpropanoid pathway, promotes osmotic stress survival in plants. In the present study, a relatively high concentration of levels of this metabolite was found in *Ocimum basilicum* in comparison to other *Ocimum* species samples. Carvacrol, a monoterpenoid phenol, is derived mainly through the methyl-erythritol-phosphate (MEP) pathway in the plastids. This molecule is known for its high antimicrobial activity. Substantial concentration of carvacrol was found in *O. kilimandscharicum*. Caftaric acid, a phenolic acid, provides plant better UVB protection [45]. In the present study, a high concentration of caftaric acid was found in *O. africanum*, *O. basilicum*, and *O. sanctum* samples. Further, the concentrations of phenolic acid metabolites coniferaldehyde (precursor of eugenol) and protocatechuic acid were found to be relatively higher in *O. sanctum* samples and Hybrid Tulsi samples, respectively, in comparison to other samples. Random forest classification classified all samples correctly with an OOB error 0 (Figure S8). Further untargeted metabolite profiling classifies species variety-specific variation of *Ocimum* samples (Figure S9).

### 3.2. GC-MS-Based Metabolic Fingerprinting Identified Ocimum Species-Specific Variation

**Figure 4:**
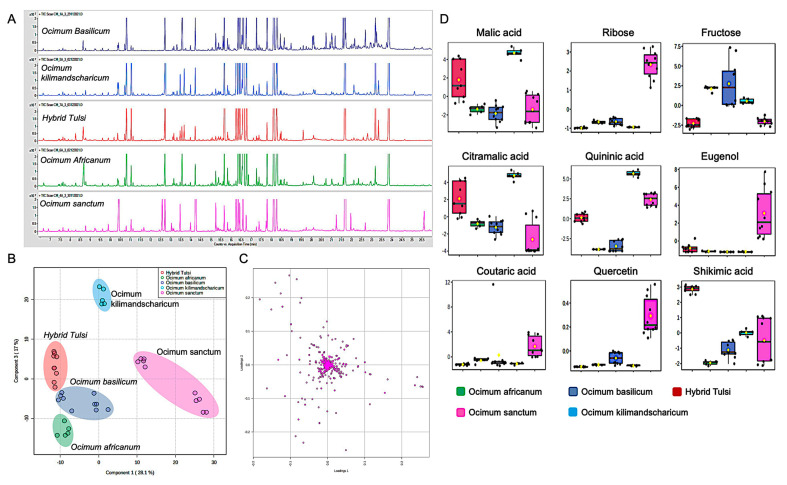
GC-MS-based metabolite fingerprinting identified species-specific differences in five different *Ocimum* species samples. (**A**) TIC of metabolite profile of five different *Ocimum* species. (**B**) PLS-DA scores plot for the first component (28.1%) vs. second component (17%), indicating differences between *Ocimum* species samples. (**C**) Corresponding loading plot for the metabolites. (**D**) Box plot for discriminatory marker metabolites, identifying the differences in five different *Ocimum* species. Normalised intensities are presented in the *y*-axis.

The GC-MS chromatographic profile of five *Ocimum* species is shown in Figure 4A. A tight clustering of QC samples explains the repeatability of the analytical system used for untargeted metabolite profiling (Figure S9A). Initially, a non-parametric multivariate analysis method, PCA, was used to project GC-MS spectra into lower dimensional space so that inherent data structure with reduced dimensional representation of original data can be revealed. The PCA model obtained revealed a general structure of the complete dataset, in which the first two principal components cumulatively accounted for 50% of the total variation, with PC1 accounting for 29.6% of the variation discriminating *O. sanctum* species samples from the other four species (Figure S9B). PC3 with 20.4 variation explains the variation of *O. basilicum* and *O. africanum* from the other two species Hybrid Tulsi and *O. kilimandscharicum*. A supervised PLS-DA was also performed to find a small number of linear combinations of the original variables that described most of the variability of metabolome of the *Ocimum* samples from five different species. As presented in Figure 4, five different clusters were identified in the PLS-DA scores plot, in which two components cumulatively accounted for 45.1% of the total variation with first component explaining (28.1%) variation between *O. sanctum* and *O. basilicum*, *O. africanum*, Hybrid Tulsi, and *O. kilimandscharicum* samples and the second component explaining the variation between Hybrid Tulsi, *O. kilimandscharicum*, *O. africanum*, and *O. basilicum* samples (Figure 4B). The corresponding loading plot that was responsible for the observed separation between *Ocimum* species samples is shown in Figure 4C. Fivefold cross-validation was further performed to find the predictive accuracy and fit of the polynomial model (Figure S11A). A permutation test was performed to assess the statistical significance of these apparently highly predictive multivariate models. For this, the supervised models were validated using 1000 permutation tests (Figure S11B). From the analysis of these distributions, the significance of the power of the optimal models to predict the metabolic profiles of sample groups was determined to be *p* < 0.001. Specific metabolites responsible for the species-specific discrimination of *Ocimum* samples were further identified using VIP values obtained from the corresponding loading plot (Table S3). Nine metabolites, in which four of them were the primary metabolites malic acid, citramalic acid, ribose, and fructose, and the other five were the secondary metabolites quininic acid, eugenol, gluconic acid, quercetin, and shikimic acid, were considered as discriminatory markers to identify species-specific *Ocimum* samples (Figure 4D). 

Malic acid, a TCA cycle intermediate, plays a crucial role in stomatal opening and closing in plant leaves [46]. Furthermore, this metabolite in mitochondria acts as a common reserve anion in plant vacuoles [46]. Citramalic acid, an analogue of malic acid, was also detected in *Ocimum* samples. This is a methyl derivative of malic acid derived from the C5-branched dibasic acid metabolism that takes place in the chloroplast stoma [46]. In the present study, a high concentration of malic acid and citramalic acid were present in *O. kilimandscharicum* in comparison to the other *Ocimum* species samples. 

Ribose is a monosaccharide produced in plant cells through the pentose phosphate pathway that is essential for ATP production [47]. In addition to its critical role in energy production, this five-carbon chain sugar is a vital component of the synthesis of biomolecules, DNA, RNA, and acetyl coenzyme A [46,47]. Supplementing ribose externally in the soil/diet enhances plant growth. Furthermore, this metabolite helps plants to incur additional stress/shock following transplantation [48]. A high concentration of ribose was found in *Ocimum sanctum* in comparison to other *Ocimum* species. Fructose, a six-membered monosaccharide, is a secondary product of plant photosynthesis after glucose. This molecule is the sweetest naturally occurring sugar, estimated to be twice as sweet as sucrose with a fruity aroma. Further, this metabolite functions as a regulatory sugar and interacts with signalling by plant hormones [49]. In the present study, a high concentration of fructose was found in *O. basilicum* samples in comparison to other species. 

Eugenol, a phenolic monoterpenoid derived from the phenylpropanoid pathway, is usually found in aromatic herbal plants [43]. Plants use this secondary metabolite as a defence molecule against microorganisms and pests, and as a floral attractant of pollinators. This molecule has high economic value as it has been widely used as an essential oil, aroma ingredient, and food flavouring [50]. In addition, this molecule exhibits effective antioxidant activity. In the present study, a high concentration of eugenol was found in *Ocimum sanctum* samples in comparison to other *Ocimum* species. Shikimic acid is a crucial metabolite in plant metabolism for the synthesis of aromatic amino acids, tyrosine, tryptophan, phenylalanine, and vitamins, and the corresponding shikimate pathway is a major link between primary and secondary metabolism, responsible for the synthesis of different secondary metabolites [46]. In the present study, a high concentration of shikimic acid was found in Hybrid Tulsi samples. Quercetin, a penta hydroxyl flavanol derived from the phenylpropanoid pathway, potently provides plants with tolerance against several abiotic and biotic stresses. In the present study, a high concentration of quercetin was found in *O. sanctum* leaf samples in comparison to other *Ocimum* species samples. Further, the random forest classification model classified samples with OOB error 0 (Figure S12). 

### 3.3. Rapid Metabolic Fingerprinting with FT-NIR Identified Species- and Variety-Specific Variation of Ocimum Samples

**Figure 5:**
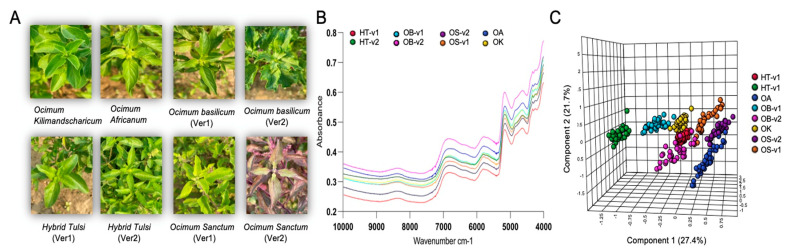
FT-NIR-based rapid metabolic fingerprinting identified species- and variety-specific variation of *Ocimum* samples. (**A**) Images of eight different *Ocimum* samples. (**B**) FT-NIR spectrum of average spectra of *Ocimum* samples from eight different varieties. (**C**) The corresponding PLS-DA plot for the first component (27.4%) vs. second component (21.7%) differentiated the *Ocimum* species and varieties.

FT-NIR is a spectroscopic technique that has been widely using in the authentication of herbal products and agricultural products, as well as in numerous natural product analyses [25,28]. However, to date, the rapid spectroscopic method combined with chemometrics has not been developed and applied to the authentication of species or varieties of *Ocimum* herbs. In this study, eight different types of *Ocimum* plant leaves, namely, *O. africanum*, *O. basilicum* variety 1 (OB-v1), *O. basilicum* variety (OB-v2), Hybrid Tulsi variety-1 (HT-v1), Hybrid Tulsi variety-2 (HT-v2), *O. kilimandscharicum* (OK), *O. sanctum* variety-1 (OS-v1), and *O. sanctum* variety-2 (OS-v2), were analysed using FT-NIR. Overall, 280 samples, with each species/variety having 35 replicates, were analysed in the range of 10,000–4000 cm^−1^ using FT-NIR spectroscopy, and the spectral profile obtained is shown in Figure 5. In all models, the best pre-processing was quantile normalisation followed by Pareto scaling. The average profiles of all samples are shown in Figure 5. The differences were essentially in the absorption intensities. FT-NIR peaks were attributed for stretching and bending vibrations that characterised the functional groups: (i) 4200–4800 cm^−1^; (ii) 4800–5250 cm^−1^; (iii) 5400–6000 cm^−1^; (iv) 6300–7200 cm^−1^; (v) 8000–8800 cm^−1^.

**Figure 6:**
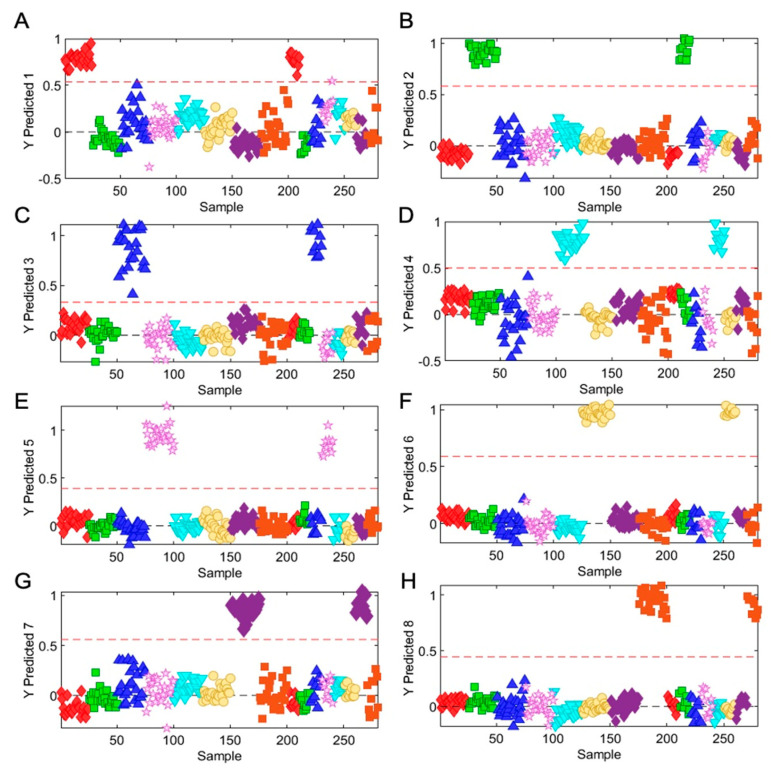
Predicted models for *Ocimum* samples by PLS-DA model. (**A**) HT-v1—Hybrid Tulsi variety 1; (**B**) HT-v2—Hybrid Tulsi variety 2; (**C**) OB-v1—*Ocimum basilicum* variety 1; (**D**) OB-v2—*Ocimum basilicum* variety 2; (**E**) OS-V1—*Ocimum sanctum* variety 1; (**F**) OS-V1—*Ocimum sanctum* variety 2; (**G**) OA—*Ocimum africanum*; (**H**) OK—*Ocimum kilimandscharicum*.

From the spectral profiles obtained, it was observed that there were spectral differences in absorbance intensities of *Ocimum* samples. Chemometric models were built to discriminate between and classify the samples according to their species/variety. Initially, a preliminary exploratory analysis of the data using PCA was employed. The unsupervised PCA model obtained from FT-NIR spectra of all samples revealed the general structure of the complete dataset, in which the first two principal components cumulatively accounted for 62.5% of the total variation, with PC1 most importantly accounting for 39% of variance, discriminating HT-v2, OS-v1, and OK from OA and OS-v2 samples (Figure S13). PC2 was responsible for 23.5% variance for discriminating OS-v2 and HT-v2 samples from all other *Ocimum* samples (Figure S13). Furthermore, supervised PLS-DA was performed additionally to find a small number of linear combinations of the original variables, which was predicted for the class membership, and that described most of the variability of the FT-NIR metabolic profile of all *Ocimum* group samples. As presented in Figure 5C, eight distinct clusters were identified in the PLS-DA scores plot, in which two components cumulatively accounted for 49.1% of the total variation, with the first component explaining 27.4% of the variation between OB-v1, OB-v2, HT-v1, HT-v2 and OS-v1, OS-v2, and OA and the second component explaining 21.7% of the variation. PLS-DA was used to validate individual models with 2/3 samples considered as the calibration set and the remaining 1/3 samples considered as the validation set (Figure 6). The PLS-DA model, having 100% sensitivity and 100% specificity with 100% accuracy and 100% reliability, was obtained for the training sets and validation sets for all samples of *Ocimum* groups. Table 1 shows the values obtained for the merit figures for the complete PLS-DA model, and Figure 6 illustrates the corresponding predictions of *Ocimum* sample groups. The calculated values for the DD-SIMCA model using FT-NIR data are presented in Table 2, with 100% sensitivity of all *Ocimum* training sample groups. For the test samples set, 100% specificity was obtained for all groups of *Ocimum* samples.

**Figure 7:**
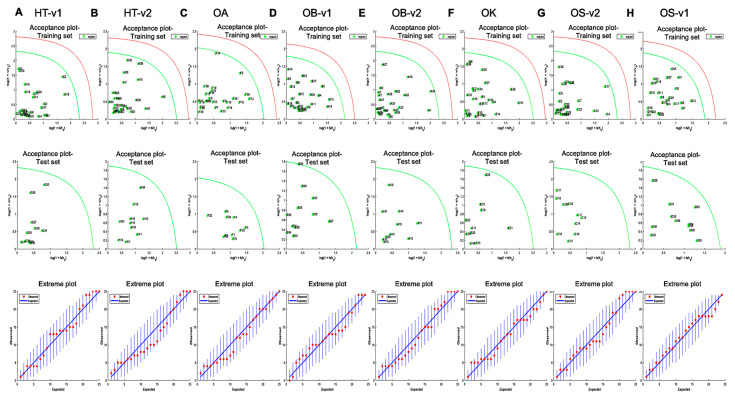
The application of DD-SIMCA for the classification of *Ocimum* samples from (**A**) HT-v1—Hybrid Tulsi variety 1; (**B**) HT-v2—Hybrid Tulsi variety 2; (**C**) OA—*O. africanum*; (**D**) OB-v1—*O. basilicum* variety 1; (**E**) OB-v2—*O. basilicum* variety 2; (**F**) OK—*O. kilimandscharicum*; (**G**) OS-V2—*O. sanctum* variety 2; (**H**) OS-V1—*O. sanctum* variety 1. Acceptance plots for training set and test set shown one to one below and the corresponding extreme plots for each training set shown far down to the acceptance plots. The acceptance plot for training set provides a graphic representation of the acceptance area, the area inside the green curve and the red line is the outlier cut-off with threshold α = 0.01. Authentic samples falling outside the green curve were considered extremes.

In the DD-SIMCA method, one class model was also used for classification. The model was used to identify species- and variety-specific *Ocimum* samples. The method consists of two-steps: Firstly, the decomposition of training data matrix by PCA and the secondly classification of new sample set with the derived principal components, represented by the acceptance area in the orthogonal distance (OD) vs. score distance (SD) as an accepted plot, with tan α value. This α value specifies a type 1 error, i.e., false negative decisions. Here, in the models, we considered performing external validation using 70% of the target class samples from each species/variety samples in the calibration set, as well as the remaining samples in the test. The models of the acceptance plots for training and test sets are shown in Figure 7. One hundred percent sensitivity and specificity were obtained for all four groups samples. The summary of DD-SIMCA performance is presented in Table 2. 

Another supervised model, K-nearest neighbours, was used for the classification of *Ocimum* samples. Initially, the complete model (built with 1557 variables obtained in the FT-NIR) did not perform well. Classification using the top 18 variables (value less than 2% of the original quantity) had better results for all *Ocimum* group samples. In this case, we considered all 280 samples for the analysis. A total of 105 of 280 samples were randomly selected for model validation, i.e., the test set. The remaining 175 samples were considered as a training set. Factor K = 16 was used for classification in the region of 7500 to 4300 cm^−1^. The model classified all samples correctly with 100% sensitivity and specificity, and there were no cases of false positives and false negatives. The corresponding results are presented in Table S4. 

Upon analysing the profiles, we noted that spectral regions related to hydroxyl (4817 cm^−1^ and 4913 cm^−1^) and C-O plus O-H combinations first overtone region (5210 to 5314 cm^−1^) were selected [51]. The variations in the absorption intensities of these regions are related to the main differences in composition between eight different varieties of *Ocimum* samples, justifying the selection of these variables.

Overall, the work presented here involves a comprehensive approach including both FT-NIR-based rapid fingerprinting and mass-spectrometry-based confirmatory methods for the identification of *Ocimum* species. The key strengths of the present work involve the rapid screening of *Ocimum* samples without any sample destruction and without the need of isolation of essential oils from *Ocimum* leaves for their species identification. Further, mass-spectrometry-based complementary approaches with minimal sample preparations for the species and variety confirmation of *Ocimum* samples are presented. Identification of *Ocimum* species in real time in the field is challenging. The present study needs to be further explored for the real-time screening of *Ocimum* leaves from the fields that would greatly help farmers. 

## 4. Conclusions

Natural medicinal herbs consist of many diversified metabolites and the classification of species and varieties, and blends are difficult to accomplish. Comprehensive metabolomic fingerprinting approaches may offer rapid and confirmatory metabolite fingerprinting for the classification of medicinal herbs. In this study, we presented for the first time the species and variety discrimination of medicinal herb *Ocimum* samples using rapid FT-NIR-based metabolic fingerprinting and LC-MS- and GC-MS-based untargeted metabolomics with the aid of chemometrics including multivariate and one-class models. The high predictive ability of models including PLS-DA, DD-SIMCA, and KNN, in which all samples were correctly classified, was demonstrated. Untargeted LC-MS-based metabolomics identified flavanoids and phenolics as a major class of metabolites that distinguished species-specific variation of *Ocimum* samples. Moreover, metabolic fingerprinting identified sub-variety classification of different *Ocimum* species. The key advantage of the comprehensive metabolic fingerprinting system is that a large number of samples can be screened using NIR-based fingerprinting and non-confirming samples can be referred to for confirmatory analysis using LC-MS-based metabolite marker analysis. The present work demonstrated that using a two-tiered system of the rapid fingerprinting method alongside a confirmatory method is appropriate to classify *Ocimum* species and varieties. The present strategy can also be used for the classification of other natural products and herbs with various species, varieties, and chemotypes.

## Figures and Tables

**Table 1:** Calculated values for the merit figures for the PLS-DA model using FT-NIR data for *Ocimum* species and varieties.

	Complete Model					
	Training					
Variety	Sen (%)	TFN (%)	Spe (%)	TFP (%)	Acc (%)	Rel (%)
HT-V1	100	0	100	0	100	100
HT-V2	100	0	100	0	100	100
OA	100	0	100	0	100	100
OB-V2	100	0	100	0	100	100
OB-V1	100	0	100	0	100	100
OK	100	0	100	0	100	100
OS-V2	100	0	100	0	100	100
OS-V1	100	0	100	0	100	100
	**TEST**					
HT-V1	100	0	100	0	100	100
HT-V2	100	0	100	0	100	100
OA	100	0	100	0	100	100
OB-V2	100	0	100	0	100	100
OB-V1	100	0	100	0	100	100
OK	100	0	100	0	100	100
OS-V2	100	0	100	0	100	100
OS-V1	100	0	100	0	100	100

Note: 70% of samples were considered as training set and 30% samples were considered as test set.

**Table 2:** Summarisation of the performance of the DD-SIMCA models for *Ocimum* variety samples.

Types	PC	α	γ	DOF (SD)	DOF (OD)	SEN	SPE
HT-v1	2	0.010	0.01	1	1	100	100
HT-v2	2	0.010	0.01	1	2	100	100
OA	2	0.010	0.01	2	2	100	100
OB-v1	2	0.010	0.01	2	3	100	100
OB-v2	2	0.010	0.01	1	1	100	100
OK	2	0.010	0.01	1	2	100	100
OS-v2	2	0.010	0.01	1	1	100	100
OS-v1	2	0.010	0.01	3	3	100	100

Note: PCs denotes the number of principal components; DoF denotes the degree of freedom; SD denotes score of distance; OD denotes orthogonal distance; SEN denotes the sensitivity of the model; SPE denotes the specificity of the model; α and γ denote type I error and outlier significance level, respectively.

## Data Availability

The data presented in this study are available in this article and in the supplementary information.

## Supplementary Materials

The following supporting information can be downloaded at https://www.mdpi.com/article/10.3390/metabo13010122/s1, Supplementary information is available alongside the article. Figures S1–S13; Tables S1–S4. Figure S1: QC plots for (A) LC-MS neg mode; (B) LC-MS pos mode. Figure S2: TIC metabolite profile *Ocimum* samples from five different species in LC-MS neg mode. Figure S3: TIC metabolite profile *Ocimum* samples from five different species in LC-MS pos mode. Figure S4: PCA plots for (A) LC-MS neg mode; (B) LC-MS pos mode. Figure S5: PLS-DA scores plot for five different species of *Ocimum* samples acquired in LC-MS-positive ionisation mode. Figure S6: PLS-DA loadings plot for the *Ocimum* species acquired in (A) LC-MS ESI (−) mode and (B) LC-MS ESI (+) mode. Figure S7: PLS-DA validation results for *Ocimum* samples analysed by LC-MS. Figure S8: Random forest classification of model *Ocimum* samples. Figure S9: Variety specific variations of *Ocimum* samples. Figure S10: QC plot and PCA plot for *Ocimum* species samples obtained by GC-MS. Figure S11: PLS-DA cross-validation results for *Ocimum* samples analysed by GC-MS. Figure S12: Random forest classification of models for *Ocimum* samples acquired using GC-MS. Figure S13: PCA analysis of FT-NIR spectral profiles of *Ocimum* samples from different species and varieties. Table S1: *Ocimum* species and the varieties used in the present study. Table S2: Discriminatory markers based on LC-MS/MS for *Ocimum* samples from different species. Table S3: Discriminatory markers for *Ocimum* samples from different species. Table S4: Classification results for test samples with K-Nearest Neighbors.

## References

[1] Bungãu S.G., Popa V.-C. (2015). Between religion and science some aspects concerning illness and healing in antiquity. Transylv. Rev..

[2] Atanasov A.G., Waltenberger B., Pferschy-Wenzig E.M., Linder T., Wawrosch C., Uhrin P., Temml V., Wang L., Schwaiger S., Heiss E.H. (2015). Discovery and Resupply of Pharmacologically Active Plant-Derived Natural Products: A Review. Biotechnol. Adv..

[3] (2000). General Guidelines for Methodologies on Research and Evaluation of Traditional Medicine.

[4] Huck C. (2015). Infrared Spectroscopic Technologies for the Quality Control of Herbal Medicines. Evidence-Based Validation of Herbal Medicine.

[5] Glevitzky I., Dumitrel G.A., Glevitzky M., Pasca B., Otrisal P., Bungau S., Cioca G., Pantis C., Popa M. (2019). Statistical Analysis of the Relationship between Antioxidant Activity and the Structure of Flavonoid Compounds. Rev. Chim..

[6] Ekor M. (2014). The Growing Use of Herbal Medicines: Issues Relating to Adverse Reactions and Challenges in Monitoring Safety. Front. Neurol..

[7] Singh D., Chaudhuri P.K. (2018). A Review on Phytochemical and Pharmacological Properties of Holy Basil (*Ocimum sanctum* L.). Ind. Crops Prod..

[8] Nahak G., Mishra R.C., Sahu R.K. (2011). Taxonomic Distribution, Medicinal Properties and Drug Development Potentiality of Ocimum (Tulsi). Drug Invent. Today.

[9] Sestili P., Ismail T., Calcabrini C., Guescini M., Catanzaro E., Turrini E., Layla A., Akhtar S., Fimognari C. (2018). The Potential Effects of Ocimum Basilicum on Health: A Review of Pharmacological and Toxicological Studies. Expert Opin. Drug Metab. Toxicol..

[10] Chaiyana W., Anuchapreeda S., Punyoyai C., Neimkhum W., Lee K.H., Lin W.C., Lue S.C., Viernstein H., Mueller M. (2019). Ocimum Sanctum Linn. as a Natural Source of Skin Anti-Ageing Compounds. Ind. Crops Prod..

[11] Abidoyea A.O., Ojedokunb F.O., Fasogbonc B.M., Bamidele O.P. (2022). Effects of Sweet Basil Leaves (*Ocimum basilicum* L.) Addition on the Chemical, Antioxidant, and Storage Stability of Roselle Calyces (*Hibiscus sabdariffa*) Drink. Food Chem..

[12] Maria Simona C., Muste S., Viman I., Georgiana F. (2020). Basil (*Ocimum basilicum* L.) Chemical Compositions and Applications in Food Processing. Hop and Medicinal Plants. https://www.researchgate.net/publication/351370972.

[13] Collin H. (2006). Herbs, Spices and Cardiovascular Disease.

[14] Dharsono H.D.A., Putri S.A., Kurnia D., Dudi D., Satari M.H. (2022). *Ocimum* Species: A Review on Chemical Constituents and Antibacterial Activity. Molecules.

[15] Da Silva Moura E., Faroni L.R.D.A., Heleno F.F., Rodrigues A.A.Z., Prates L.H.F., De Queiroz M.E.L.R. (2020). Optimal Extraction of *Ocimum basilicum* Essential Oil by Association of Ultrasound and Hydrodistillation and Its Potential as a Biopesticide against a Major Stored Grains Pest. Molecules.

[16] Chowdhury T., Mandal A., Roy S.C., De Sarker D. (2017). Diversity of the Genus *Ocimum* (Lamiaceae) through Morpho-Molecular (RAPD) and Chemical (GC–MS) Analysis. J. Genet. Eng. Biotechnol..

[17] Balekundri A., Mannur V. (2020). Quality Control of the Traditional Herbs and Herbal Products: A Review. Futur. J. Pharm. Sci..

[18] Yeshi K., Crayn D., Ritmejerytė E., Wangchuk P. (2022). Plant Secondary Metabolites Produced in Response to Abiotic Stresses Has Potential Application in Pharmaceutical Product Development. Molecules.

[19] Tangpao T., Charoimek N., Teerakitchotikan P., Leksawasdi N., Jantanasakulwong K., Rachtanapun P., Seesuriyachan P., Phimolsiripol Y., Chaiyaso T., Ruksiriwanich W. (2022). Volatile Organic Compounds from Basil Essential Oils: Plant Taxonomy, Biological Activities, and Their Applications in Tropical Fruit Productions. Horticulturae.

[20] Avetisyan A., Markosian A., Petrosyan M., Sahakyan N., Babayan A., Aloyan S., Trchounian A. (2017). Chemical Composition and Some Biological Activities of the Essential Oils from Basil *Ocimum* Different Cultivars. BMC Complement. Altern. Med..

[21] Gurav T.P., Dholakia B.B., Giri A.P. (2022). A Glance at the Chemodiversity of *Ocimum* Species: Trends, Implications, and Strategies for the Quality and Yield Improvement of Essential Oil. Phytochem. Rev..

[22] Patel M.K., Pandey S., Kumar M., Haque M.I., Pal S., Yadav N.S. (2021). Plants Metabolome Study: Emerging Tools and Techniques. Plants.

[23] Lee S., Oh D.G., Singh D., Lee J.S., Lee S., Lee C.H. (2020). Exploring the Metabolomic Diversity of Plant Species across Spatial (Leaf and Stem) Components and Phylogenic Groups. BMC Plant Biol..

[24] Ch R., Chevallier O., McCarron P., McGrath T.F., Wu D., Nguyen Doan Duy L., Kapil A.P., McBride M., Elliott C.T. (2021). Metabolomic Fingerprinting of Volatile Organic Compounds for the Geographical Discrimination of Rice Samples from China, Vietnam and India. Food Chem..

[25] Villate A., San Nicolas M., Gallastegi M., Aulas P.A., Olivares M., Usobiaga A., Etxebarria N., Aizpurua-Olaizola O. (2021). Review: Metabolomics as a Prediction Tool for Plants Performance under Environmental Stress. Plant Sci..

[26] Emwas A.H., Roy R., McKay R.T., Tenori L., Saccenti E., Nagana Gowda G.A., Raftery D., Alahmari F., Jaremko L., Jaremko M. (2019). Nmr Spectroscopy for Metabolomics Research. Metabolites.

[27] Salem M.A., De Souza L.P., Serag A., Fernie A.R., Farag M.A., Ezzat S.M., Alseekh S. (2020). Metabolomics in the Context of Plant Natural Products Research: From Sample Preparation to Metabolite Analysis. Metabolites.

[28] Beć K.B., Grabska J., Huck C.W. (2021). NIR Spectroscopy of Natural Medicines Supported by Novel Instrumentation and Methods for Data Analysis and Interpretation. J. Pharm. Biomed. Anal..

[29] Muráriková A., Ťažký A., Neugebauerová J., Planková A., Jampílek J., Mučaji P., Mikuš P. (2017). Characterization of Essential Oil Composition in Different Basil Species and Pot Cultures by a GC-MS Method. Molecules.

[30] Rastogi S., Shah S., Kumar R., Kumar A., Shasany A.K. (2020). Comparative Temporal Metabolomics Studies to Investigate Interspecies Variation in Three Ocimum Species. Sci. Rep..

[31] Pandey R., Kumar B. (2016). HPLC-QTOF-MS/MS-Based Rapid Screening of Phenolics and Triterpenic Acids in Leaf Extracts of Ocimum Species and Their Interspecies Variation. J. Liq. Chromatogr. Relat. Technol..

[32] Bhuvaneshwari K., Gokulanathan A., Jayanthi M., Govindasamy V., Milella L., Lee S., Yang D.C., Girija S. (2016). Can *Ocimum basilicum* L. and *Ocimum tenuiflorum* L. in Vitro Culture Be a Potential Source of Secondary Metabolites?. Food Chem..

[33] Ratti C. (2001). Hot Air and Freeze-Drying of High-Value Foods: A Review. J. Food Eng..

[34] Ch R., Rey G., Ray S., Jha P.K., Driscoll P.C., Dos Santos M.S., Malik D.M., Lach R., Weljie A.M., MacRae J.I. (2021). Rhythmic Glucose Metabolism Regulates the Redox Circadian Clockwork in Human Red Blood Cells. Nat. Commun..

[35] Pomerantsev A.L., Rodionova O.Y. (2014). Concept and Role of Extreme Objects in PCA/SIMCA. J. Chemom..

[36] Westerhuis J.A., Hoefsloot H.C.J., Smit S., Vis D.J., Smilde A.K., Velzen E.J.J., Duijnhoven J.P.M., Dorsten F.A. (2008). Assessment of PLSDA Cross Validation. Metabolomics.

[37] Hastie T., Tibshirani R., Friedman J. (2017). Springer Series in Statistics the Elements of Statistical Learning Data Mining, Inference, and Prediction.

[38] Koo I., Kim S., Zhang X. (2013). Comparative Analysis of Mass Spectral Matching-Based Compound Identification in Gas Chromatography-Mass Spectrometry. J. Chromatogr. A.

[39] Granato D., Putnik P., Kovačević D.B., Santos J.S., Calado V., Rocha R.S., Da Cruz A.G., Jarvis B., Rodionova O.Y., Pomerantsev A. (2018). Trends in Chemometrics: Food Authentication, Microbiology, and Effects of Processing. Compr. Rev. Food Sci. Food Saf..

[40] Shannon M., Ratnasekhar C.H., McGrath T.F., Kapil A.P., Elliott C.T. (2021). A Two-Tiered System of Analysis to Tackle Rice Fraud: The Indian Basmati Study. Talanta.

[41] Falcone Ferreyra M.L., Rius S.P., Casati P. (2012). Flavonoids: Biosynthesis, Biological Functions, and Biotechnological Applications. Frontiers in Plant Science. Front. Res. Found..

[42] Khalid M., Saeed-ur-Rahman, Bilal M., Huang D. (2019). Role of Flavonoids in Plant Interactions with the Environment and against Human Pathogens—A Review. Journal of Integrative Agriculture. Chin. Acad. Agric. Sci..

[43] Prinsi B., Morgutti S., Negrini N., Faoro F., Espen L. (2020). Insight into Composition of Bioactive Phenolic Compounds in Leaves and Flowers of Green and Purple Basil. Plants.

[44] Majdi M., Malekzadeh-Mashhady A., Maroufi A., Crocoll C. (2017). Tissue-Specific Gene-Expression Patterns of Genes Associated with Thymol/Carvacrol Biosynthesis in Thyme (*Thymus vulgaris* L.) and Their Differential Changes upon Treatment with Abiotic Elicitors. Plant Physiol. Biochem..

[45] Fu R., Zhang P., Deng Z., Jin G., Zhang Y. (2021). Chicoric Acid Provides Better Ultraviolet Protection than the Sum of Its Substrates in Purple Coneflower Plants. Ind. Crops Prod..

[46] Daloso D.M., Medeiros D.B., dos Anjos L., Yoshida T., Araújo W.L., Fernie A.R. (2017). Metabolism within the Specialized Guard Cells of Plants. New Phytol..

[47] Riggs J.W., Rockwell N.C., Cavales P.C., Callis J. (2016). Identification of the Plant Ribokinase and Discovery of a Role for Arabidopsis Ribokinase in Nucleoside Metabolism. J. Biol. Chem..

[48] Seifert J.G., Shecterle L.M. (2013). Use of Ribose to Enhance Plant Growth. U.S. Patent.

[49] Cho Y.H., Yoo S.D. (2011). Signaling Role of Fructose Mediated by FINS1/FBP in Arabidopsis Thaliana. PLoS Genet..

[50] Khalil A.A., Rahman U.U., Khan M.R., Sahar A., Mehmood T., Khan M. (2017). Essential Oil Eugenol: Sources, Extraction Techniques and Nutraceutical Perspectives. RSC Advances. R. Soc. Chem..

[51] Ma L., Peng Y., Pei Y., Zeng J., Shen H., Cao J., Qiao Y., Wu Z. (2019). Systematic Discovery about NIR Spectral Assignment from Chemical Structural Property to Natural Chemical Compounds. Sci. Rep..

