# Supplementary material for: Comprehensive Metabolomic Fingerprinting Combined with Chemometrics Identifies Species- and Variety-Specific Variation of Medicinal Herbs: An Ocimum Study
Source: Metabolites. 2023 Jan 13;13(1):122. doi: 10.3390/metabo13010122 (PMC9862730; doi:10.3390/metabo13010122)
Supplement: Supplementary file 1 [file metabolites-13-00122-s001.zip › metabolites-2124482-supplementary.pdf]

Supplementary Material

(Figure S1-Figure S13; Table S1-Table S4)

A

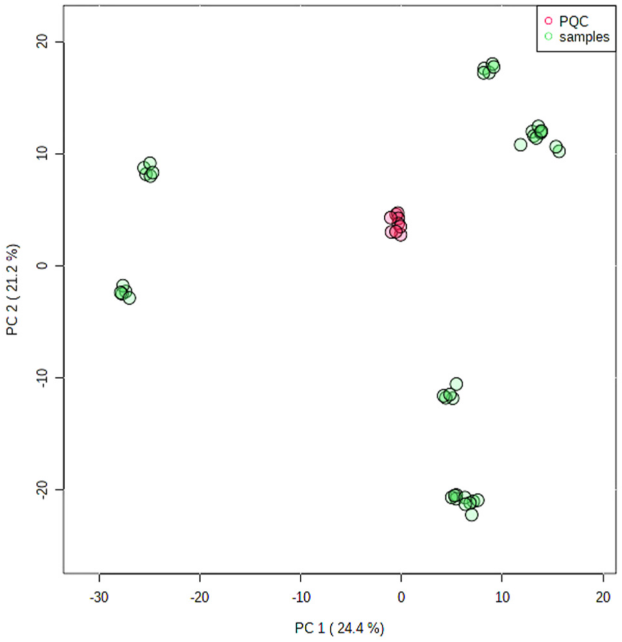

B

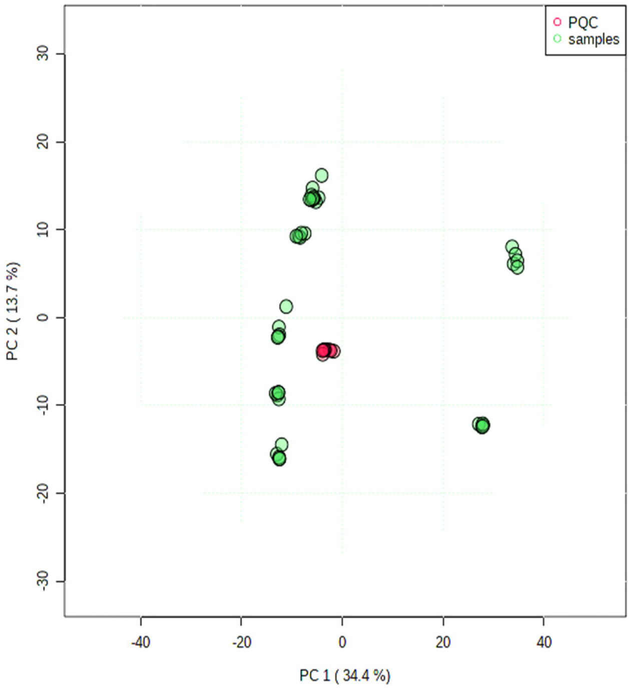

Figure S1: QC plots for A) LC-MS neg mode B) LC-MS pos mode

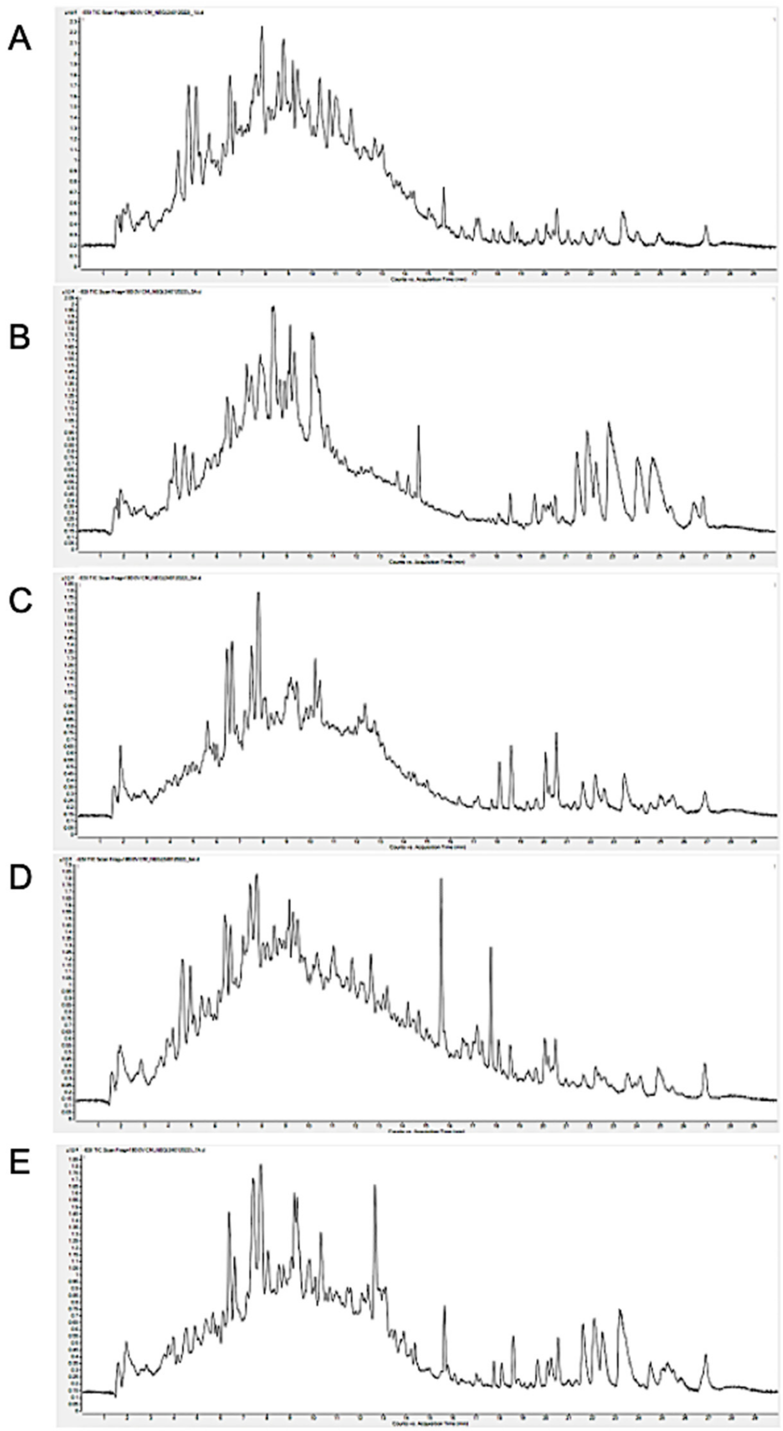

Figure S2: TIC metabolites profile LC-MS neg mode.

A Ocimum basilicum
B Ocimum sanctum
C Ocimum kilimandscharicum
D Ocimum africanum
E Hybrid Tulsi

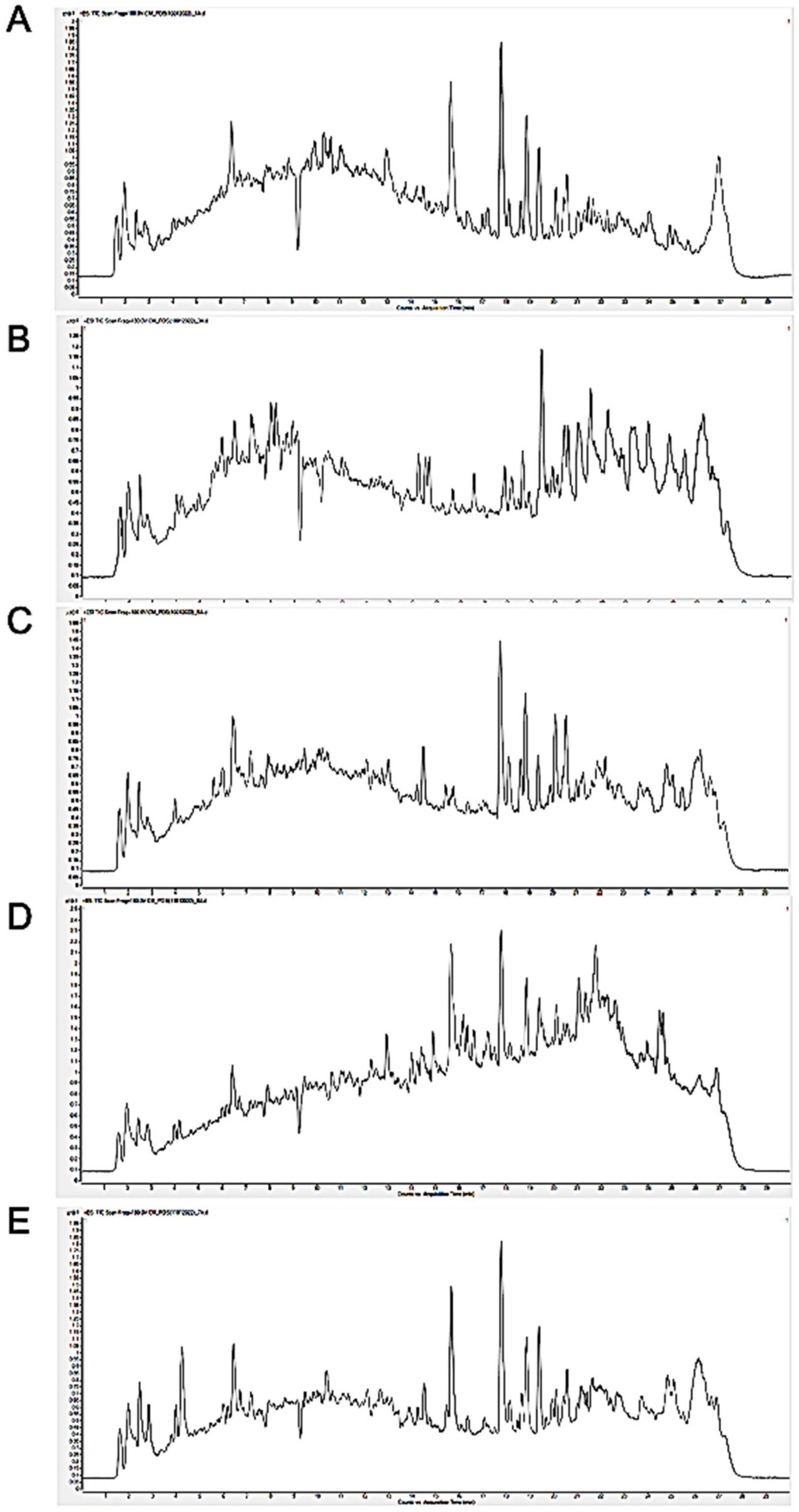

Figure S3: TIC metabolites profile LC-MS pos mode.

A Ocimum basilicum
B Ocimum sanctum
C Ocimum kilimandscharicum
D Ocimum africanum
E Hybrid Tulsi

A

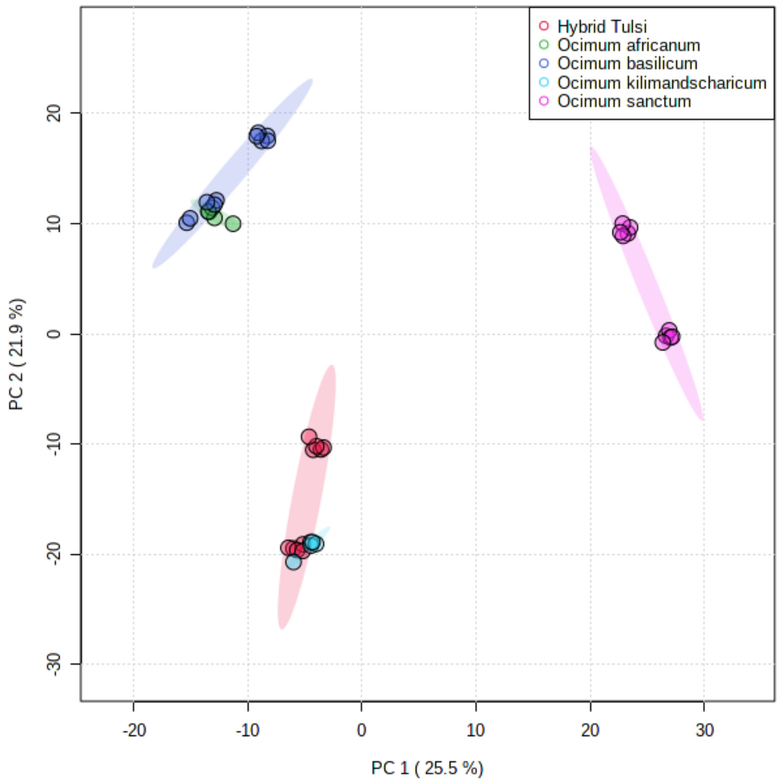

B

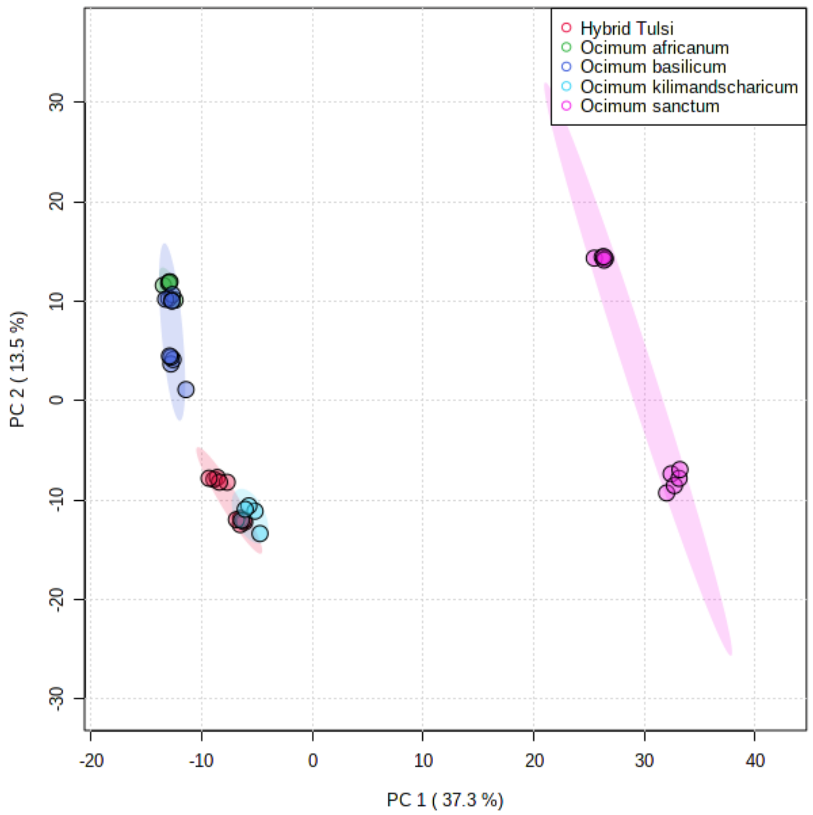

Figure S4: PCA plots for A) LC-MS neg mode B) LC-MS pos mode

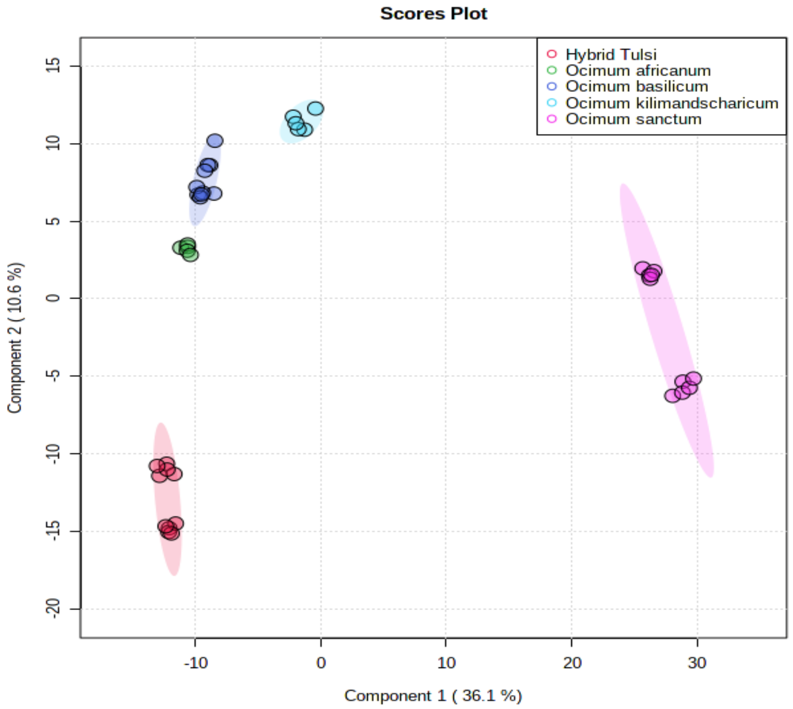

Figure S5: PLS-DA scores plot for five different species of *Ocimum* samples acquired in LC-MS positive ionization mode.

A) B)

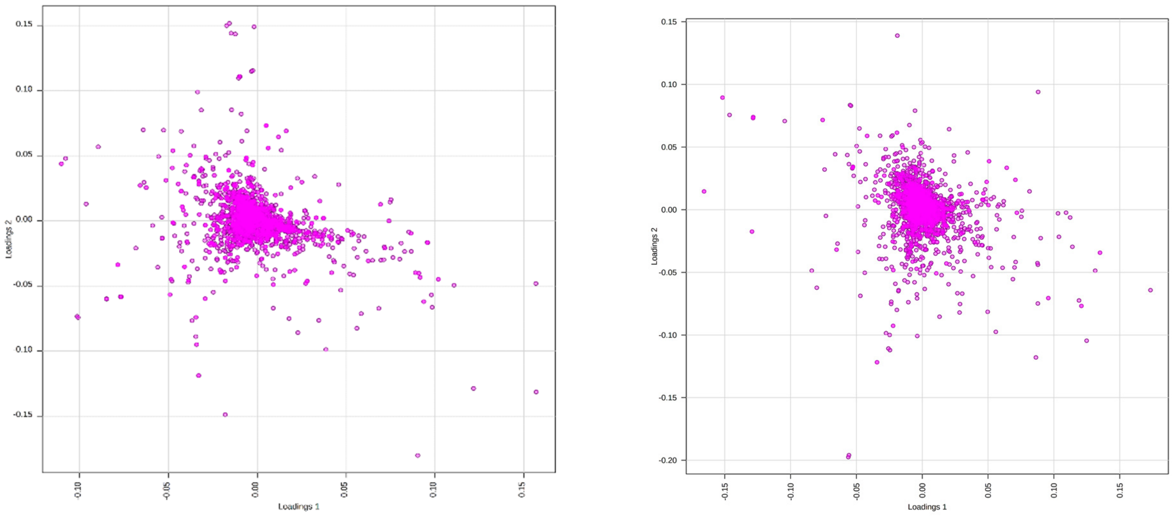

Figure S6: PLS-DA loadings plot for the *Ocimum* species acquired in A) LC-MS ESI (-) mode and B) LC-MS ESI (+) mode respectively.

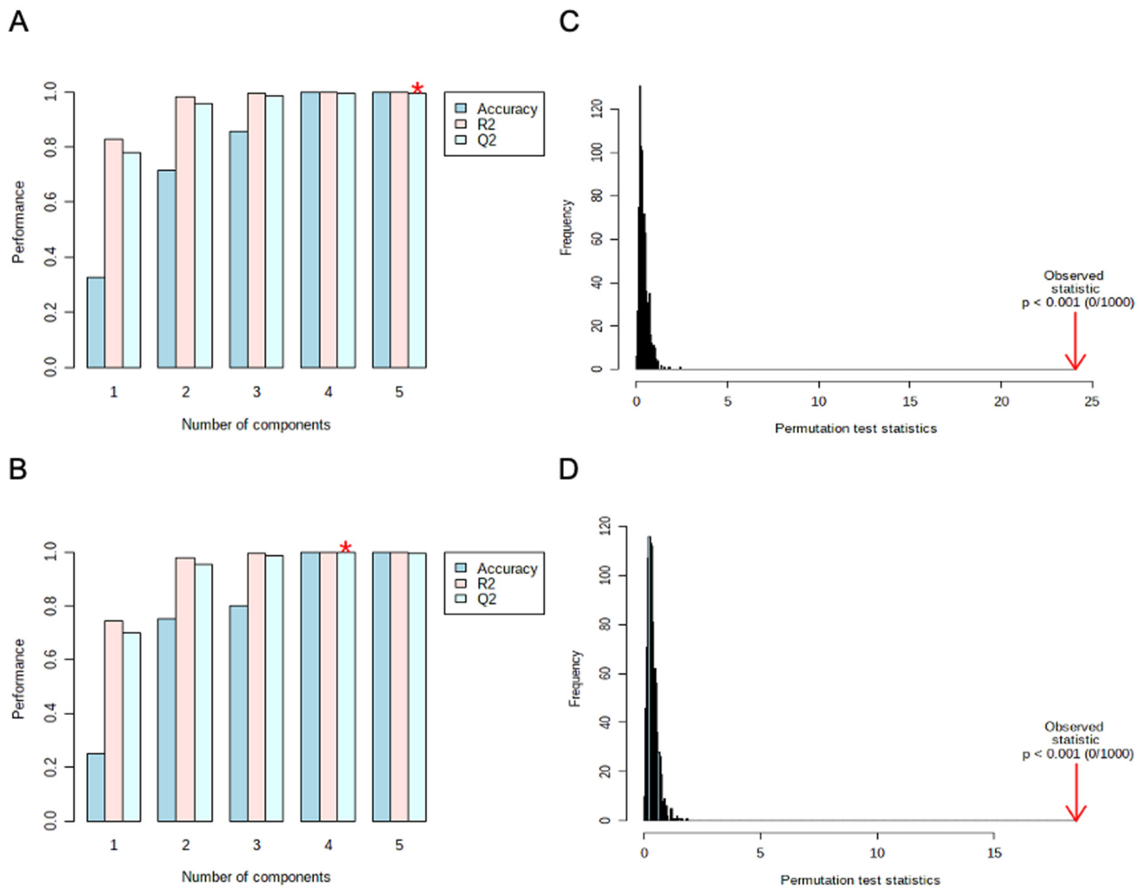

Figure S7: PLS-DA Validation results for *Ocimum* samples analysed by LC-MS A) & B) Multivariate analysis using PLS-DA cross validation for LC-MS based metabolite profile acquired in ESI (-) mode and ESI (+) mode respectively. Bar plots showing the three performance measures (prediction accuracy, R^2 and Q^2) using different number of components. The red '*' indicates the best values of the currently selected measures (Q^2). C) & D) Statistical validation of the PLS-DA by permutation analysis using 1000 different model permutations. The goodness of fit and predictive capability of the original class assignments is much higher compared to ratios based on the permutation class assignments ($P < 0.001$).

A

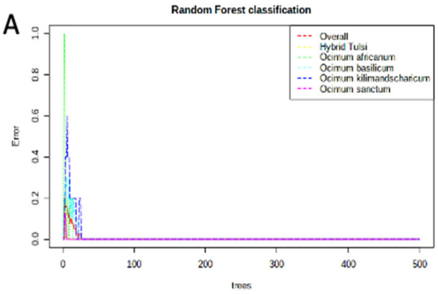

The OOB error is 0.0

	Hybrid Tulsi	Ocimum africanum	Ocimum basilicum	Ocimum kilimandscharicum	Ocimum sanctum	class.error
Hybrid Tulsi	10	0	0.0	0.0	0.0	0.0
Ocimum africanum	0	5	0.0	0.0	0.0	0.0
Ocimum basilicum	0	0	10.0	0.0	0.0	0.0
Ocimum kilimandscharicum	0	0	0.0	5.0	0.0	0.0
Ocimum sanctum	0	0	0.0	0.0	10.0	0.0

B

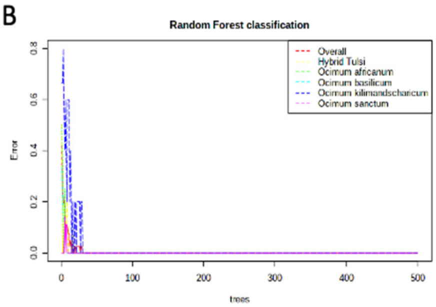

The OOB error is 0.0

	Hybrid Tulsi	Ocimum africanum	Ocimum basilicum	Ocimum kilimandscharicum	Ocimum sanctum	class.error
Hybrid Tulsi	10	0	0.0	0.0	0.0	0.0
Ocimum africanum	0	5	0.0	0.0	0.0	0.0
Ocimum basilicum	0	0	10.0	0.0	0.0	0.0
Ocimum kilimandscharicum	0	0	0.0	5.0	0.0	0.0
Ocimum sanctum	0	0	0.0	0.0	10.0	0.0

Figure S8: Random Forest classification of models *Ocimum* samples A) Metabolite profile acquired by LC-MS ESI (-) mode, B) Metabolite profile acquired by LC-MS ESI (+) mode

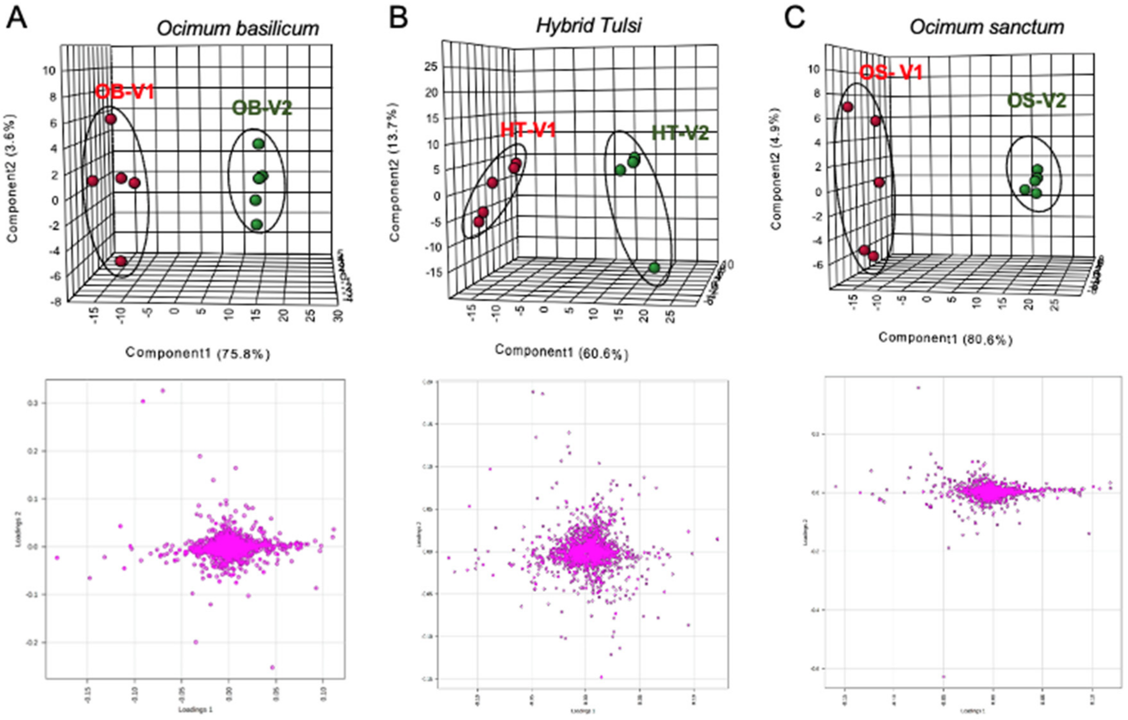

Figure S9: Variety specific variation of *Ocimum* samples from A) *Ocimum basilicum* B) Hybrid Tulsi C) *Ocimum sanctum*

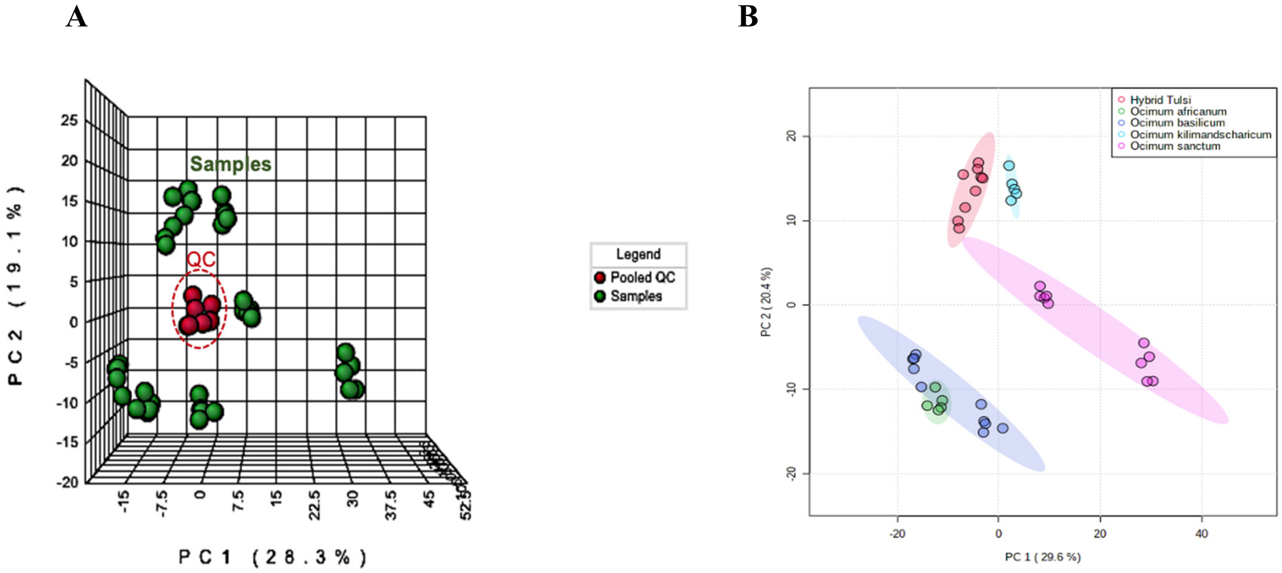

Figure S10: A) PCA plot for GC-MS based metabolite profile of Quality control samples and *Ocimum* samples. B) PCA plot for *Ocimum* species samples acquired through GC-MS based metabolite profiling. PC1 explains 29.6% variation and PC2 explains 20.4% variation.

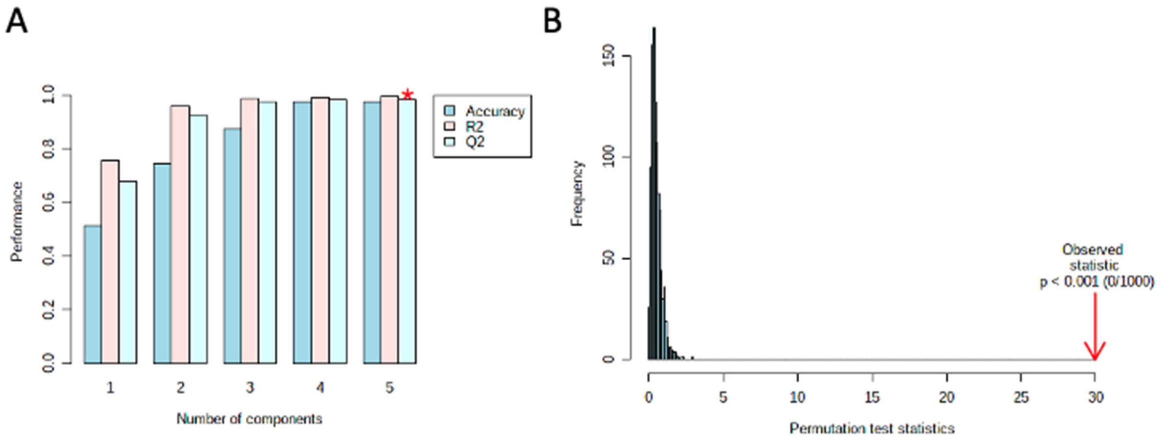

Figure S11: A) Multivariate analysis using PLS-DA cross validation. Bar plots showing the performance measures, prediction accuracy, R^2 and Q^2, using different number of components. The red '*' indicates the best values of the currently selected measures (Q^2). B) Statistical validation of the PLS-DA by permutation analysis using 1000 different model permutations. The goodness of fit and predictive capability of the original class assignments is much higher compared to ratios based on the permutation class assignments ($P < 0.001$).

	Hybrid Tulsi	Ocimum africanum	Ocimum basilicum	Ocimum kilimandscharicum	Ocimum sanctum	class.error
Hybrid Tulsi	9	0	0.0	0.0	0.0	0.0
Ocimum africanum	0	5	0.0	0.0	0.0	0.0
Ocimum basilicum	0	0	10.0	0.0	0.0	0.0
Ocimum kilimandscharicum	0	0	0.0	5.0	0.0	0.0
Ocimum sanctum	0	0	0.0	0.0	10.0	0.0

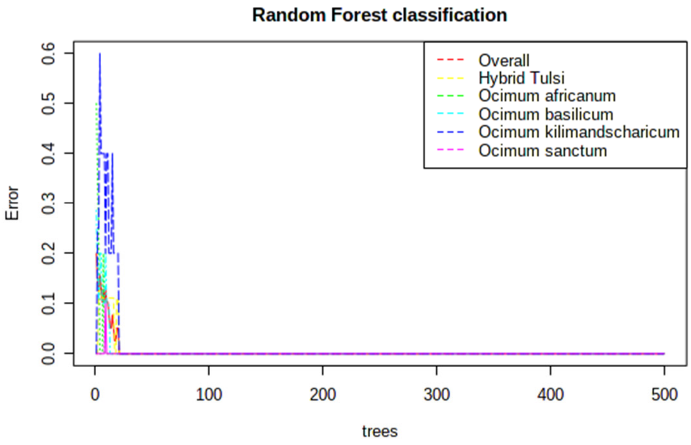

Figure S12: Random Forest classification of models for *Ocimum* samples acquired using GC-MS.

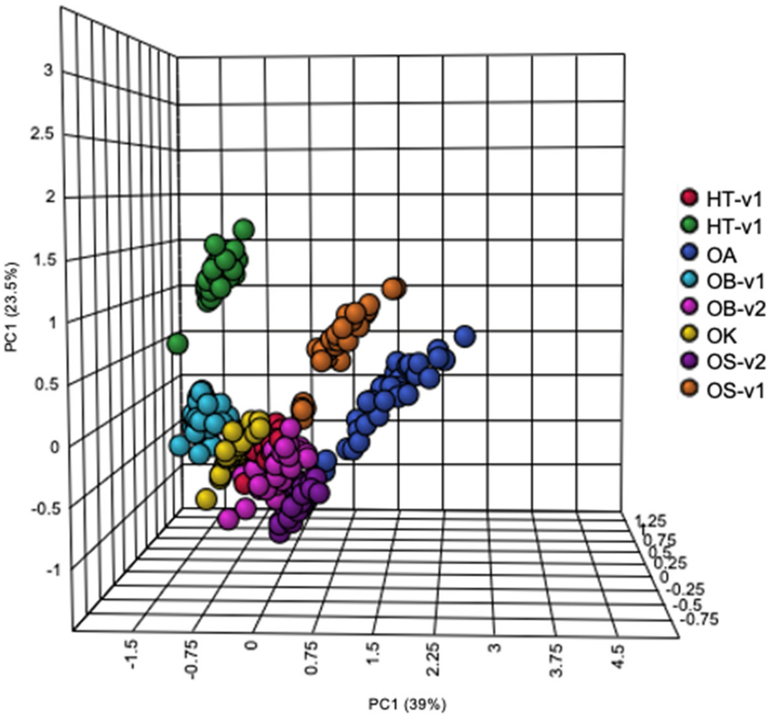

Figure S13: PCA analysis of FT-NIR spectral profiles of *Ocimum* samples from different species and varieties.

Table S1: Ocimum species and the varieties used in the present study.

S.NO	Ocimum species	Variety
1	*Ocimum basilicum*	OB-v1
		OB-v2
2	*Ocimum Sanctum*	OS-v1
		OS-v2
3	*Ocimum africanum*	*Ocimum africanum*
4	*Ocimum* Hybrid Tulsi	HT-v1
		HT-v2
5	*Ocimum kilimandscharicum*	*Ocimum kilimandscharicum*

Province India, CSIR-CIMAP Lucknow

Table S2: Discriminatory markers based on LC-MS/MS for *Ocimum* samples from different species.

Metabolite	Formulae	VIP	Ionization mode	Adduct	ppm	m/z	RT	Pubchem ID	P value	FDR	MS/MS
Cpd 106: Kaempferol-3-O-rutinoside	C27 H30 O15	2.733	Pos	M+H	4.28	595.1631	8.144	5318767	1.2004E-15	1.6066E-14	287.0550, 449.10785,269.0450, 431.0978, 449.1084,147.0657
20Cpd 19: Apigenin-7-O-glucuronide	C21 H18 O11	3.865	Pos	M+H	-0.68	447.0923	8.922	12912214	4.7188E-36	8.2107E-34	271.0602, 253.0501, 241.0501, 177.0399
Cpd 88: Gardenin B	C19 H18 O7	2.579	Pos	M+H	0.48	359.1123	18.879	96539	2.1599E-20	6.2636E-19	344.0890, 316.0941
Cpd 103: Kaempferol	C15 H10 O6	2.070	Pos	M+H	-0.06	287.0549	10.882	5280863	1.7363E-20	6.0423E-19	269.0450, 165.05463, 257.0450,245.0450
Cpd 55: Cirsilineol	C18 H16 O7	1.410	Pos	M+H	0.14	345.0969	9.462	162464	8.6581E-6	2.1833E-5	330.0734,315.0869,329.0661,313.0712,285.0763
Cpd 17: Apigenin	C15 H10 O5	1.598	Pos	M+H	0.64	271.0599	12.154	5280443	1.3656E-5	3.2109E-5	253.0501,241.0501,229.0501
Cpd 158: Rosmarinic acid	C18 H16 O8	2.036	Pos	M+H	0.27	361.0916	13.022	5281792	1.3185E-4	2.5211E-4	163.03897,181.04953,145.02841, 117.03349,
Cpd 142: Carvacrol	C10 H14 O	3.272	Pos	M+H	0.45	151.1117	8.944	10364	3.4225E-16	4.9626E-15	91.0542,93.0699,107.0491,43.0542,81.0699,79.0542
Cpd 166: Salvianolic acid C	C26 H20 O10	4.857	Pos	M+H	0.17	493.1128	12.594	13991590	3.6157E-8	1.6131E-7	295.0601,267.0652,465.1180,475.1024,447.1074, 385.0918
Cpd 33: Caftaric acid	C13 H12 O9	2.170	Neg	M-H	2.36	311.04	4.637	6440397	1.3425E-13	9.4561E-13	135.0452,103.0037,161.0244,133.0295,105.0193,
Cpd 151: Protocatechuic acid	C7 H6 O4	2.301	Pos	M+H	2.67	155.0339	4.101	72	2.4537E-22	1.0674E-20	137.0239,109.0290,111.0446,125.0239,107.0133, 125.0239,79.0184
Cpd 78: Genkwanin	C16 H12 O5	1.153	Neg	M-H	2.04	283.061	15.506	5281617	1.4665E-10	5.9395E-10	242.05736,270.05228,167.03389
Cpd 69: confieraldehyde	C10 H10 O3	1.704	Pos	M+H	-0.85	179.0701	20.96	5280536	5.3268E-6	1.4043E-5	161.0603,149.0663,55.0184,147.0446,137.0603,43.0184,131.0497,125.0603

Table S3: Discriminatory markers for *Ocimum* samples from different species.

Compound	Rt	Formulae	VIP	P-Value	FDR	m/z	PubChemID
Malic acid, 3TMS	12.72	C13H30O5Si3	1.132	9.6887E-10	4.687E-9	73,147,233,245,133	525
D-Ribose, 4TMS	15.24	C17H42O5Si4	2.378	6.7304E-20	3.8094E-18	73,217,204,191,147	10975657
D-(-)-Fructose, pentakis(trimethylsilyl) ether, methyloxime	16.43	C22H55NO6Si5	2.481	2.4845E-8	9.6317E-8	73,103,217,147,307	5984
Citramalic acid, 3TMS	12.59	C14H32O5Si3	2.368	3.6699E-10	1.8546E-9	73,247,147,259,115	1081
Quininic acid (5TMS)	16.26	C22H52O6Si5	3.515	3.4102E-25	6.4339E-23	73,345,147,255,191	345824
Eugenol TMS	12.47	C13H20O2Si	2.869	2.0791E-7	6.8416E-7	206,236,205,207,221	3314
Gulonic acid, .gamma.-lactone, 4TMS derivavative	15.6	C18H42O6Si4	1.953	5.4385E-12	3.6214E-11	73,147,217,103,204	165105
Quercetin, 5TMS	24.96	C30H50O7Si5	3.068	3.6039E-12	2.5183E-11	647,73,648,649,559	5280343
Shikimic acid, 4TMS	15.68	C19H42O5Si4	1.946	3.2173E-12	2.3051E-11	73,204,147,205,206	8742

Table S4: Classification results for test samples with k-Nearest Neighbors

OCM_test	HTSh	HTSu	OA	OB V1	OB V2	OK	Os V2	OS V1	Row Total
TSh	12<br>1.000<br>1.000<br>0.114	0<br>0.000<br>0.000<br>0.000	0<br>0.000<br>0.000<br>0.000	0<br>0.000<br>0.000<br>0.000	0<br>0.000<br>0.000<br>0.000	0<br>0.000<br>0.000<br>0.000	0<br>0.000<br>0.000<br>0.000	0<br>0.000<br>0.000<br>0.000	12<br>0.114
HTSu	0<br>0.000<br>0.000<br>0.000	14<br>1.000<br>1.000<br>0.133	0<br>0.000<br>0.000<br>0.000	0<br>0.000<br>0.000<br>0.000	0<br>0.000<br>0.000<br>0.000	0<br>0.000<br>0.000<br>0.000	0<br>0.000<br>0.000<br>0.000	0<br>0.000<br>0.000<br>0.000	14<br>0.133
OA	0<br>0.000<br>0.000<br>0.000	0<br>0.000<br>0.000<br>0.000	13<br>1.000<br>1.000<br>0.124	0<br>0.000<br>0.000<br>0.000	0<br>0.000<br>0.000<br>0.000	0<br>0.000<br>0.000<br>0.000	0<br>0.000<br>0.000<br>0.000	0<br>0.000<br>0.000<br>0.000	13<br>0.124
OB Sh	0<br>0.000<br>0.000<br>0.000	0<br>0.000<br>0.000<br>0.000	0<br>0.000<br>0.000<br>0.000	13<br>1.000<br>1.000<br>0.124	0<br>0.000<br>0.000<br>0.000	0<br>0.000<br>0.000<br>0.000	0<br>0.000<br>0.000<br>0.000	0<br>0.000<br>0.000<br>0.000	13<br>0.124
OB Sm	0<br>0.000<br>0.000<br>0.000	0<br>0.000<br>0.000<br>0.000	0<br>0.000<br>0.000<br>0.000	0<br>0.000<br>0.000<br>0.000	13<br>1.000<br>1.000<br>0.124	0<br>0.000<br>0.000<br>0.000	0<br>0.000<br>0.000<br>0.000	0<br>0.000<br>0.000<br>0.000	13<br>0.124
OK	0<br>0.000<br>0.000<br>0.000	0<br>0.000<br>0.000<br>0.000	0<br>0.000<br>0.000<br>0.000	0<br>0.000<br>0.000<br>0.000	0<br>0.000<br>0.000<br>0.000	14<br>1.000<br>1.000<br>0.133	0<br>0.000<br>0.000<br>0.000	0<br>0.000<br>0.000<br>0.000	14<br>0.133
Os Ang	0<br>0.000<br>0.000<br>0.000	0<br>0.000<br>0.000<br>0.000	0<br>0.000<br>0.000<br>0.000	0<br>0.000<br>0.000<br>0.000	0<br>0.000<br>0.000<br>0.000	0<br>0.000<br>0.000<br>0.000	14<br>1.000<br>1.000<br>0.133	0<br>0.000<br>0.000<br>0.000	14<br>0.133
OS Ayu	0	0	0	0	0	0	0	12	12

	0.000<br>0.000<br>0.000	0.000<br>0.000<br>0.000	0.000<br>0.000<br>0.000	0.000<br>0.000<br>0.000	0.000<br>0.000<br>0.000	0.000<br>0.000<br>0.000	0.000<br>0.000<br>0.000	1.000<br>1.000<br>0.114	0.114
Column Total	12<br>0.114	14<br>0.133	13<br>0.124	13<br>0.124	13<br>0.124	14<br>0.133	14<br>0.133	12<br>0.114	105

Total number of samples = 280

Total number of Training samples set = 175; Test samples set = 105

i. 12 out of 35 observations were accurately predicted as HT-V1 constitutes of 11.4%
ii. 14 out of 35 observations were accurately predicted as HT-V2 constitutes of 13.3%
iii. 13 out of 35 observations were accurately predicted as OA constitute of 12.4%
iv. 13 out of 35 observations were accurately predicted as OB-V1 constitute of 12.4%
v. 11 out of 35 observations were accurately predicted as OB-V2 constitute of 12.4%
vi. 11 out of 35 observations were accurately predicted as OK constitute of 13.3%
vii. 11 out of 35 observations were accurately predicted as OS-V2 constitute of 13.3%
viii. 11 out of 35 observations were accurately predicted as OS-V1 constitute of 11.4%

There are no cases of false positives and false negatives.

Total accuracy of model = 100%
